# Effect of variety and malting conditions on proteolytic activity, free amino nitrogen, and soluble protein contents of two maize varieties (*Atp-Y* and *Coca-sr*): amylolytic activity and physico-chemical and functional properties of optimal sample

**DOI:** 10.3389/fnut.2023.1163915

**Published:** 2023-08-07

**Authors:** Stephano Tambo Tene, Oluwafemi Ayodeji Adebo, Derek Tantoh Ndinteh, Anthony Olusegun Obilana, Hermann Arantes Kohole Foffe, Justine Odelonne Kenfack, Michael Hermann Kengne Kamdem, Julie Mathilde Klang, Hilaire Macaire Womeni

**Affiliations:** ^1^Research Unit of Biochemistry of Medicinal Plants, Food Sciences and Nutrition, Department of Biochemistry, Faculty of Science, University of Dschang, Dschang, Cameroon; ^2^Department of Biotechnology and Food Technology, Faculty of Science, University of Johannesburg Doornfontein Campus, Johannesburg, Gauteng, South Africa; ^3^Centre for Natural Products Research, Department of Chemical Sciences, University of Johannesburg Doornfontein Campus, Johannesburg, Gauteng, South Africa; ^4^Department of Food Science and Technology, Cape Peninsula University of Technology, Bellville, South Africa

**Keywords:** *Coca-sr*, *Atp-Y*, proteolytic activity, FAN, soluble protein, soaking, germination, maturation

## Abstract

**Introduction:**

The utilization of sprouted meals in beer production and enhancing the physicochemical properties of supplementary foods is widespread in Africa. This work aimed to determine the influence of soaking, germination, maturation and variety conditions on the physicochemical properties, proteolytic activity, free amino nitrogen (FAN) and soluble protein contents of *Coca-sr* and *Atp-Y* maize varieties.

**Methods:**

To achieve this, the central composite design (CCD) was used for the optimization of five parameters, namely soaking time (18–42 h), plant salt concentration (0.5–1.2%), soaking temperature (25–41°C), sprouting time (80–195 h) and ripening time (17.50–42 h), and following dependent variables were investigated: proteolytic activity, FAN content and soluble protein. Optimal samples flours obtained were then subsequently subjected to physicochemical and functional analysis.

**Results:**

The analysis of results showed that the linear, interactive and quadratic effects of the factors significantly (*p*<0.05) affected the proteolytic activity, FAN and soluble protein contents of both varieties. The direction of each factor's variation and its effects were not similar in the two varieties. The optimal malting conditions were 7.31 h soaking with 1.678% vegetable salt at a temperature of 34.65°C followed by sprouting for 245.59 h and maturation for 0.765 h for the *Atp-Y* variety. For the *Coca-sr* variety, it requires 1.608 h of soaking with 1.678% vegetable salt at a temperature of 51.93°C followed by 273.94 h and 58.73 h for sprouting and ripening time respectively. The meals of *Coca-sr* produces using these optimal conditions showed a significantly (*p*<0.05) higher proteolytic activity, FAN and soluble protein content. The amylolytic activity was more pronounced in the *Atp-Y* variety, as was the content of essential amino acids. The above optimal conditions reduced the content of anti-nutrients (phytates, saponins, oxalates, condensed and hydrolysable tannins), improved the availability of minerals (Ca and Mg), reduced the pH, mass density, water retention capacity and swelling rate.

**Conclusion:**

As a result, the optimal flours of these two maize varieties could be applied in the formulation of supplementary foods, bakery products and beer by industrialists.

## 1. Introduction

Cereals play an important role in the diet of people in developing countries. Among these cereals, maize is one of the most important after rice and wheat, with an annual production estimated at more than 1.6 million tons (1). It is an important source of carbohydrates, energy, minerals such as calcium, sodium, and potassium, and vitamins such as vitamin B1 (2). However, the utilization of these nutrients, especially the digestibility of macronutrients such as lipids, carbohydrates, and proteins, is impacted by several factors such as the presence of antinutrients such as phytates, oxalates, trypsin inhibitors, tannins, lectins, and saponins that can complex these nutrients leading to the formation of indigestible complexes (3). There is an urgent need to apply treatments that will improve these parameters. Some ancient technologies such as fermentation, boiling, roasting, and sprouting have been shown to positively impact the physicochemical and nutritional characteristics of cereals (2).

Malting could be a solution to this phenomenon because, according to one study, it reduces anti-nutritional factors, improves the digestibility of different macronutrients, increases the content of certain nutrients such as proteins, and improves the quality of amino acids, as well as the sensory properties of the flours (4–6). During this process, the seed absorbs water during soaking, which is necessary for the hydrolytic enzymes of the seeds to break their dormancy during germination. Among these hydrolytic enzymes are mainly amylases, lipases, and proteases, which are of paramount importance in the brewing, infant food, bakery, pharmaceutical, and paper industries (7). In infant feeding, amylases break down starch into simple sugars, thereby reducing the viscosity of foods and promoting sweetness (8, 9). As for proteases, they allow the digestion of proteins into amino acids, thus facilitating their digestibility. In the brewing industry, reducing sugars from the action of amylases is used in the fermentation process for the production of alcohol, while free amino acids (FANs) from the action of proteases are used as sources of energy and raw material in the synthesis of aromatic odor compounds (7).

The use of malted maize in the production of traditional alcoholic and non-alcoholic beers such as Sha'a and bili-bili (Cameroon) is known (7, 10). It is also used as a barley adjuvant by some Cameroonian breweries. Nevertheless, its full use in local beer production has not yet been reported, unlike other cereals such as sorghum and millet, which have already been tested. This is due to the quality of its malt, which unlike the others does not yield well, has a low FAN content, and low proteolytic enzymes. This problem with the quality of maize malt results in a significant loss of foreign exchange due to the import of malted barley from Europe. The quality of traditionally produced maize malt is unknown, as are the process steps, which are difficult to standardize, and there is little research in this area (7). Moreover, the interest of maize in this research also lies in the fact that it is less rich in lipids compared to other possible cereal alternatives to barley.

Analysis of malt quality including antioxidant content, FAN, and proteolytic activity shows that it is affected by several factors such as grain variety and malting conditions (7, 11, 12). To solve this problem, the response surface methodology (RSM) will be implemented in the search for optimal conditions for the production of good quality maize malt from *Atp-Y* and *Coca-sr* varieties with the best proportions of FAN, soluble protein, and good proteolytic activity. The objective of this study was, therefore, to evaluate the influence of variety, soaking conditions, germination, and maturation times on proteolytic activity, free amino nitrogen, soluble protein, and physico-chemical and functional properties of two maize varieties (*Atp-Y* and *Coca-sr*).

## 2. Materials and methods

### 2.1. Material

#### 2.1.1. Plant material

The plant used for this study consisted of two maize varieties (*Zea mays* L), *Atp-Y* and *Coca-sr* obtained from the multipurpose station of the Institute of Agricultural Research for Development (IRAD) in Dschang, Cameroon. The plant ash used was obtained as described by Tambo et al. (13).

#### 2.1.2. Reagents

Several chemical reagents were used: hydrochloric acid (HCl), sulfuric acid, 3,5-dinitrosalicylic acid (DNS), sodium hydroxide pellets (NaOH), catechechin, gallic acid, bovine serum albumin (BSA), trichloroacetic acid (TCA), ninhydrin, azocasein, phosphate, glycine, calcium chloride (Cacl_2_), albumin, vanillin, nitric acid, acetonitrile, methanol, and acetic acid. All these reagents were purchased from local stores.

### 2.2. Methods

#### 2.2.1. Production of vegetable ash

Vegetable salt was produced using the method described by Tambo et al. (13). In fact, banana peels were sun-dried for 7 days and the drying peels obtained were incinerated in a muffle furnace (Model Perkin Elmer, USA) calibrated at 300°C for 5 h. The residues were ground in an ordinary mill and then stored in polyethylene bags. The mineral composition of the plantain peel ash was assessed (data not shown).

#### 2.2.2. Optimization of the processing conditions for malted maize meal (*Atp-Y* and *Coca-sr* varieties)

##### 2.2.2.1. Production of malted maize meals

**Figure 1:**
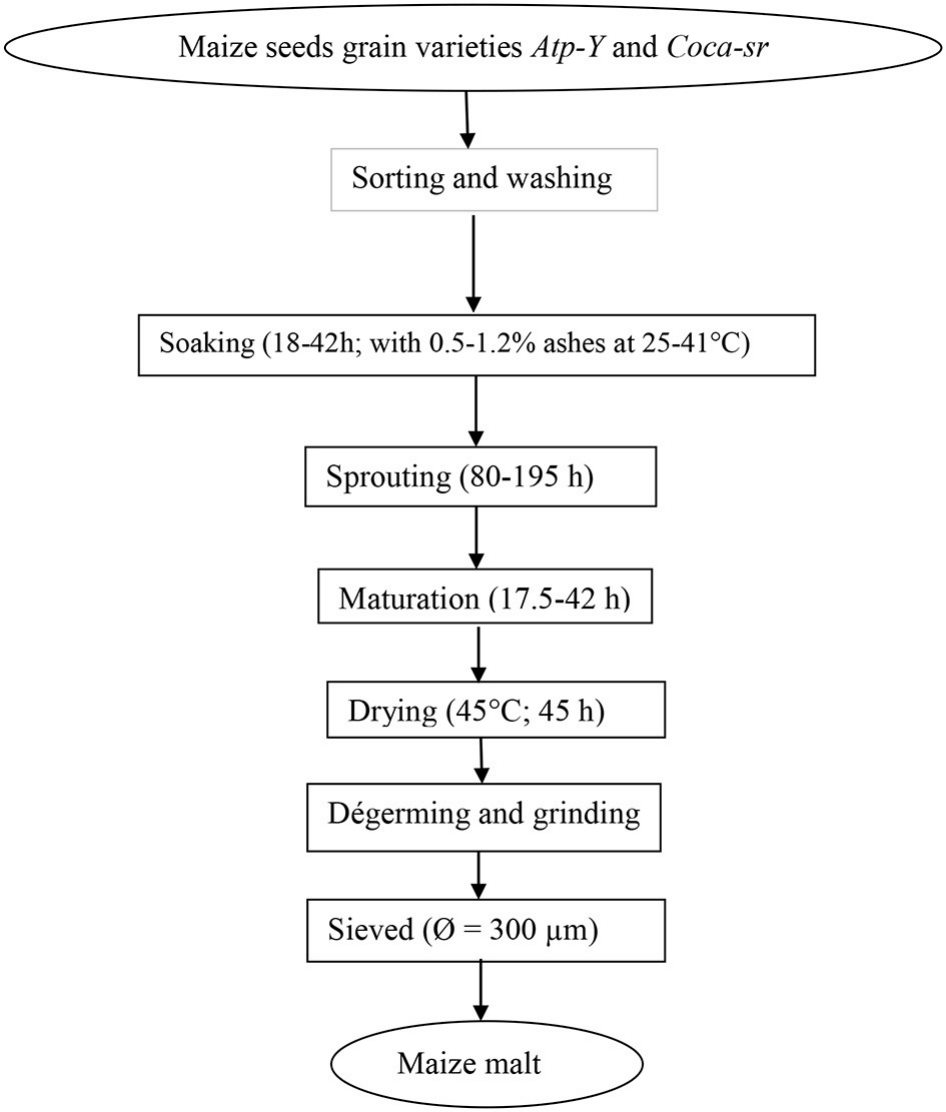
Process of transformation of maize grains into malted maize meal.

The partially modified method described by Traoré et al. (14) was followed to prepare maize meals. The same factors and conditions described by Tambo et al. (13) were used to optimize the production of sprouting maize meal. The diagram of production is presented below (Figure 1).

##### 2.2.2.2. Selection of the optimization design, factors, experimental domain, and responses

The description done by Tambo et al. (13) was used to select the design, experimental domain, and responses. The Response Surface Methodology (RSM) was used to optimize the five selected factors with the central composite design as the optimization scheme using Minitab 18.0. This central composite design applies to these five factors to generate a matrix of 54 trials (divided into 32 factorial trials, 10 in the stars and 12 in the center of the domain) as shown in Table 1.

**Table 1:** Experimental matrix, and experimental and predicted values of free amino acids, soluble proteins, and proteolytic activity according to the different trials and variety.

**N°**	**Real and coded (values in brackets) variables**					***Atp-Y*** **experimental values**			***Coca-sr*** **experimental values**			***Coca-sr*** **predicted values**			***Atp-Y*** **predicted values**		
	**X**_1_ **(h)**	**X**_2_ **(%)**	**X**_3_ **(**°**C)**	**X**_4_ **(h)**	**X**_5_ **(h)**	**FAN**	**Soluble protein**	**Proteolytic activity**	**FAN**	**Soluble protein**	**Proteolytic activity**	**FAN**	**Soluble protein**	**Proteolytic activity**	**FAN**	**Soluble protein**	**Proteolytic activity**
1	30.00 (0)	0.85 (0)	33.00 (0)	137.50 (0)	0.77 (–α)	431.55 ± 2.47^aA^	2.84 ± 0.06^aB^	0.12 ± 0.00^aA^	220.66 ± 2.08^aB^	6.15 ± 0.06^aA^	0.10 ± 0.00^aB^	210.62^b^	5.82^b^	0.10^a^	397.13^b^	2.28^b^	0.12^a^
2	30.00 (0)	0.85 (0)	33.00 (0)	137.50 (0)	58.75 (+α)	255.13 ± 2.13^bA^	4.22 ± 0.02^aB^	0.09 ± 0.00^bA^	244.23 ± 9.14^bA^	4.40 ± 0.78^aA^	0.07 ± 0.00^aB^	280.49^a^	4.89^a^	0.07^a^	303.94^a^	3.71^b^	0.11^a^
3	58.39 (+α)	0.85 (0)	33.00 (0)	137.50 (0)	29.75 (0)	189.28 ± 9.97^aB^	2.60 ± 0.32^aB^	0.08 ± 0.00^aB^	340.66 ± 13.86^bA^	3.94 ± 0.65^aA^	0.10 ± 0.00^aA^	374.09^a^	3.92^a^	0.10^a^	178.46^b^	1.88^b^	0.08^a^
4	30.00 (0)	0.85 (0)	33.00 (0)	137.50 (0)	29.75 (0)	189.40 ± 2.13^aB^	4.22 ± 2.63^aB^	0.10 ± 0.00^aA^	318.34 ± 12.73^aA^	6.15 ± 0.26^aA^	0.11 ± 0.00^aA^	322.88^a^	5.00^b^	0.11^a^	188.32^a^	4.34^a^	0.10^a^
5	30.00 (0)	0.85 (0)	33.00 (0)	137.50 (0)	29.75 (0)	170.75 ± 7.02^bB^	4.49 ± 0.25^aB^	0.10 ± 0.00^aA^	318.34 ± 0.00^bA^	4.77 ± 0.52^aA^	0.11 ± 0.00^aA^	322.88^a^	5.00^a^	0.11^a^	188.32^a^	4.34^b^	0.10^a^
6	30.00 (0)	0.85 (0)	33.00 (0)	137.50 (0)	29.75 (0)	170.75 ± 9.99^bB^	4.17 ± 0.40^aA^	0.10 ± 0.00^aA^	318.34 ± 6.37^aA^	4.50 ± 0.00^bA^	0.11 ± 0.00^aA^	322.88^a^	5.00^a^	0.11^a^	188.32^a^	4.34^a^	0.10^a^
7	30.00 (0)	0.85 (0)	14.07 (–α)	137.50 (0)	29.75 (0)	150.01 ± 0.00^bB^	4.63 ± 0.30^aB^	0.08 ± 0.00^aA^	302.94 ± 8.02^aA^	5.14 ± 0.39^aA^	0.02 ± 0.00^bB^	289.47^b^	4.85^b^	0.05^a^	171.25^a^	4.02^b^	0.07^b^
8	30.00 (0)	0.85 (0)	51.93 (+α)	137.50 (0)	29.75 (0)	72.93 ± 19.78^aB^	1.92 ± 0.05^aB^	0.02 ± 0.00^bB^	279.40 ± 3.51^bA^	3.99 ± 0.45^aA^	0.04 ± 0.00^aA^	319.09^a^	4.44^a^	0.01^b^	66.09^b^	1.47^b^	0.04^a^
9	30.00 (0)	0.85 (0)	33.00 (0)	137.50 (0)	29.75 (0)	170.75 ± 5.29^bB^	5.41 ± 0.42^aA^	0.09 ± 0.00^bB^	316.41 ± 9.96^aA^	4.77 ± 0.45^aB^	0.11 ± 0.00^aA^	322.88^a^	5.00^a^	0.11^a^	188.32^a^	4.34^b^	0.10^a^
10	30.00 (0)	0.85 (0)	33.00 (0)	273.55 (+α)	29.75 (0)	138.48 ± 5.26^bB^	9.35 ± 0.87^aA^	0.11 ± 0.00^bA^	307.85 ± 5.28^bA^	7.52 ± 1.17^aB^	0.09 ± 0.00^aB^	323.08^a^	7.70^a^	0.08^b^	156.88^a^	9.05^b^	0.12^a^
11	30.00 (0)	0.85 (0)	33.00 (0)	1.46 (–α)	29.75 (0)	175.53 ± 2.45^aB^	7.01 ± 0.00^aA^	0.04 ± 0.00^aB^	204.38 ± 13.55^aA^	2.75 ± 0.58^aB^	0.10 ± 0.00^bA^	215.37^a^	2.73^a^	0.11^a^	171.53^a^	6.25^b^	0.03^b^
12	1.61 (–α)	0.85 (0)	33.00 (0)	137.50 (0)	29.75 (0)	123.36 ± 6.36^bB^	3.85 ± 0.03^aA^	0.10 ± 0.00^bA^	364.95 ± 1.76^aA^	3.49 ± 0.65^aA^	0.10 ± 0.00^aA^	357.74^b^	3.67^a^	0.10^a^	148.58^a^	3.51^b^	0.11^a^
13	30.00 (0)	1.678 (+α)	33.00 (0)	137.50 (0)	29.75 (0)	153.98 ± 1.06^bB^	5.73 ± 1.10^aA^	0.05 ± 0.00^aB^	303.63 ± 9.56^bA^	5.18 ± 0.06^aB^	0.09 ± 0.00^aA^	326.10^a^	4.85^b^	0.07^b^	172.15^a^	4.73^b^	0.04^b^
14	30.00 (0)	0.022 (–α)	33.00 (0)	137.50 (0)	29.75 (0)	244.88 ± 9.94^aB^	4.95 ± 0.00^aB^	0.07 ± 0.00^bA^	288.01 ± 1.98^aA^	5.23 ± 0.06^bA^	0.02 ± 0.00^bB^	291.77^a^	5.73^a^	0.04^a^	241.11^a^	4.89^a^	0.08^a^
15	42.00 (+1)	1.20 (+1)	25.00 (−1)	80.00 (−1)	17.50 (−1)	209.99 ± 29.24^bA^	2.94 ± 0.17^aB^	0.02 ± 0.00^bB^	235.42 ± 4.19^bA^	3.03 ± 0.39^bA^	0.10 ± 0.00^aA^	244.68^a^	3.69^a^	0.10^a^	227.76^a^	2.79^b^	0.04^a^
16	18.00 (−1)	1.20 (+1)	41.00 (+1)	80.00 (−1)	17.50 (−1)	193.57 ± 16.62^aB^	2.47 ± 0.94^aA^	0.06 ± 0.00^bB^	260.09 ± 6.67^aA^	2.57 ± 0.32^aA^	0.14 ± 0.00^aA^	266.43^b^	2.82^a^	0.11^b^	170.20^b^	2.96^a^	0.07^a^
17	42.00 (+1)	1.20 (+1)	41.00 (+1)	195.00 (+1)	42.00 (+1)	179.82 ± 14.05^aB^	2.75 ± 0.25^bB^	0.07 ± 0.00^bA^	347.69 ± 12.26^bA^	6.79 ± 0.13^bA^	0.04 ± 0.00^bB^	363.20^a^	7.19^a^	0.05^a^	158.58^b^	3.23^a^	0.08^a^
18	18.00 (−1)	1.20 (+1)	25.00 (−1)	195.00 (+1)	42.00 (+1)	249.02 ± 22.19^aB^	6.38 ± 0.26^bA^	0.09 ± 0.00^aB^	316.05 ± 11.71^bA^	4.77 ± 0.52^aB^	0.10 ± 0.00^aA^	346.57^a^	5.15^a^	0.09^b^	236.64^b^	6.96^a^	0.08^b^
19	42.00 (+1)	0.50 (−1)	41.00 (+1)	80.00 (−1)	17.50 (−1)	260.53 ± 18.65^bA^	3.94 ± 0.74^aA^	0.04 ± 0.00^aB^	277.43 ± 5.26^aA^	3.39 ± 0.65^aA^	0.08 ± 0.00^bA^	276.44^a^	3.75^a^	0.09^a^	279.55^a^	3.47^b^	0.04^a^
20	18.00 (−1)	0.50 (−1)	41.00 (+1)	80.00 (−1)	17.50 (−1)	170.72 ± 21.06^bB^	2.66 ± 1.01^aA^	0.04 ± 0.00^bA^	272.32 ± 8.43^aA^	3.12 ± 0.45^aA^	0.03 ± 0.00^bB^	264.57^b^	3.53^a^	0.04^a^	202.32^a^	2.94^a^	0.06^a^
21	18.00 (−1)	1.20 (+1)	41.00 (+1)	80.00 (−1)	42.00 (+1)	97.68 ± 3.56^bB^	2.34 ± 0.00^bB^	0.08 ± 0.00^aB^	285.98 ± 5.50^bA^	4.12 ± 0.91^aA^	0.11 ± 0.00^bA^	311.61^a^	4.25^a^	0.12^a^	108.23^a^	2.70^a^	0.07^b^
22	18.00 (−1)	1.20 (+1)	25.00 (−1)	80.00 (−1)	17.50 (−1)	240.51 ± 13.76^aA^	2.52 ± 0.49^bB^	0.07 ± 0.00^aB^	185.99 ± 3.55^aB^	3.62 ± 0.97^aA^	0.08 ± 0.00^aA^	175.85^b^	3.72^a^	0.08^a^	230.29^b^	3.11^a^	0.07^a^
23	18.00 (−1)	0.50 (−1)	25.00 (−1)	80.00 (−1)	42.00 (+1)	246.10 ± 0.71^aB^	6.24 ± 0.76^aA^	0.06 ± 0.00^bB^	295.21 ± 5.20^bA^	5.14 ± 0.00^aB^	0.08 ± 0.00^aA^	306.89^a^	4.91^b^	0.08^a^	223.33^b^	5.09^b^	0.07^a^
24	30.00 (0)	0.85 (0)	33.00 (0)	137.50 (0)	29.75 (0)	200.36 ± 6.36^aB^	4.31 ± 0.42^aB^	0.10 ± 0.00^aB^	316.41 ± 10.57^aA^	5.04 ± 0.39^aA^	0.11 ± 0.00^aA^	322.88^a^	5.00^a^	0.11^a^	188.32^b^	4.34^a^	0.10^a^
25	30.00 (0)	0.85 (0)	33.00 (0)	137.50 (0)	29.75 (0)	200.36 ± 20.23^aB^	4.50 ± 0.37^aA^	0.09 ± 0.00^bB^	318.34 ± 11.60^aA^	4.68 ± 0.26^bA^	0.11 ± 0.00^aA^	322.88^a^	5.00^a^	0.11^a^	188.32^b^	4.34^b^	0.10^a^
26	30.00 (0)	0.85 (0)	33.00 (0)	137.50 (0)	29.75 (0)	194.95 ± 17.67^aB^	4.63 ± 0.82^aA^	0.11 ± 0.00^aA^	322.98 ± 6.69^aA^	4.04 ± 0.26^bA^	0.11 ± 0.00^aA^	322.88^a^	5.00^a^	0.11^a^	188.32^b^	4.34^b^	0.10^b^
27	42.00 (+1)	1.20 (+1)	25.00 (−1)	195.00 (+1)	17.50 (−1)	233.88 ± 18.34^aB^	3.12 ± 0.70^bB^	0.08 ± 0.00^aA^	301.30 ± 11.58^aA^	5.32 ± 1.56^aA^	0.06 ± 0.00^aB^	303.66^a^	5.65^a^	0.06^a^	218.71^b^	4.11^a^	0.06^b^
28	42.00 (+1)	0.50 (−1)	25.00 (−1)	80.00 (−1)	42.00 (+1)	253.53 ± 15.85^bB^	5.04 ± 0.68^aA^	0.10 ± 0.00^aB^	324.66 ± 8.39^bA^	4.31 ± 0.26^aB^	0.13 ± 0.00^aA^	341.83^a^	4.12^b^	0.11^b^	271.13^a^	5.39^a^	0.10^a^
29	42.00 (+1)	0.50 (−1)	41.00 (+1)	80.00 (−1)	42.00 (+1)	219.30 ± 22.25^aB^	2.94 ± 0.23^bA^	0.06 ± 0.00^aA^	294.58 ± 10.26^aA^	2.20 ± 0.32^aA^	0.05 ± 0.00^aB^	265.40^b^	2.16^a^	0.04^b^	205.89^b^	4.11^a^	0.05^b^
30	18.00 (−1)	0.50 (−1)	25.00 (−1)	195.00 (+1)	17.50 (−1)	160.51 ± 11.72^aB^	5.73 ± 0.28^aA^	0.14 ± 0.00^aA^	291.28 ± 23.98^aA^	5.05 ± 0.00^bB^	0.08 ± 0.00^aB^	288.96^b^	5.55^a^	0.07^b^	171.42^a^	5.76^a^	0.14^a^
31	42.00 (−1)	0.50 (−1)	25.00 (−1)	195.00 (+1)	17.50 (−1)	217.96 ± 3.53^bB^	3.76 ± 0.66^aB^	0.10 ± 0.00^bB^	310.81 ± 1.78^aA^	7.20 ± 0.06^bA^	0.12 ± 0.00^aA^	307.48^b^	7.40^a^	0.12^a^	227.65^a^	3.73^a^	0.13^a^
32	30.00 (0)	0.85 (0)	33.00 (0)	137.50 (0)	29.75 (0)	190.78 ± 1.78^aB^	4.45 ± 0.71^aA^	0.10 ± 0.00^aA^	336.51 ± 2.45^aA^	5.41 ± 0.26^aB^	0.10 ± 0.00^bA^	322.88^b^	5.00^b^	0.11^a^	188.32^b^	4.34^b^	0.10^a^
33	18.00 (−1)	1.20 (+1)	25.00 (−1)	195.00 (+1)	17.50 (−1)	232.93 ± 0.36^bB^	7.02 ± 0.71^aA^	0.10 ± 0.00^bA^	264.37 ± 5.86^bA^	5.23 ± 0.32^aB^	0.07 ± 0.00^aB^	276.48^a^	4.34^b^	0.06^b^	241.75^a^	6.39^b^	0.11^a^
34	18.00 (−1)	0.50 (−1)	41.00 (+1)	195.00 (+1)	42.00 (+1)	148.77 ± 2.83^bB^	4.86 ± 1.20^aA^	0.10 ± 0.00^aA^	287.32 ± 1.77^bA^	4.95 ± 0.58^aA^	0.04 ± 0.00^bB^	310.82^a^	5.49^a^	0.05^a^	154.26^a^	4.25^b^	0.09^b^
35	18.00 (−1)	1.20 (+1)	41.00 (+1)	195.00 (+1)	17.50 (−1)	226.04 ± 7.04^aB^	5.77 ± 1.20^aA^	0.14 ± 0.00^aA^	378.40 ± 17.67^aA^	4.40 ± 0.00^bB^	0.07 ± 0.00^bB^	377.94^a^	5.96^a^	0.11^a^	218.11^b^	5.51^b^	0.13^b^
36	30.00 (0)	0.85 (0)	33.00 (0)	137.50 (0)	29.75 (0)	185.15 ± 15.61^aB^	3.90 ± 0.42^aB^	0.12 ± 0.00^aA^	322.30 ± 5.72^aA^	4.86 ± 0.71^aA^	0.11 ± 0.00^aB^	322.88^a^	5.00^a^	0.11^a^	188.32^a^	4.34^a^	0.10^b^
37	18.00 (−1)	0.50 (−1)	25.00 (−1)	80.00 (−1)	17.50 (−1)	248.38 ± 9.21^bA^	2.75 ± 0.64^aB^	0.12 ± 0.00^aA^	246.82 ± 1.07^bA^	6.24 ± 0.06^aA^	0.09 ± 0.00^aB^	254.21^a^	5.92^b^	0.08^b^	265.12^a^	3.27^a^	0.09^b^
38	30.00 (0)	0.85 (0)	33.00 (0)	137.50 (0)	29.75 (0)	183.90 ± 12.38^aB^	4.40 ± 0.61^aA^	0.10 ± 0.00^aB^	334.20 ± 4.59^aA^	4.82 ± 0.45^aA^	0.11 ± 0.00^aA^	322.88^b^	5.00^a^	0.11^a^	188.32^a^	4.34^b^	0.10^a^
39	42.00 (+1)	1.20 (+1)	41.00 (+1)	80.00 (−1)	42.00 (+1)	92.19 ± 21.61^aB^	3.94 ± 0.53^aA^	0.06 ± 0.00^aA^	320.61 ± 15.16^aA^	2.38 ± 0.71^aB^	0.06 ± 0.00^bA^	306.90^b^	2.62^a^	0.07^a^	77.26^b^	3.35^b^	0.06^a^
40	18.00 (−1)	1.20 (+1)	25.00 (−1)	80.00 (−1)	42.00 (+1)	185.39 ± 17.76^aB^	4.12 ± 0.45^aA^	0.06 ± 0.00^bB^	274.57 ± 13.64^aA^	4.31 ± 0.26^aA^	0.09 ± 0.00^aA^	259.51^b^	4.43^a^	0.09^a^	171.26^b^	4.38^a^	0.07^a^
41	30.00 (0)	0.85 (0)	33.00 (0)	137.50 (0)	29.75 (0)	186.28 ± 12.46^aB^	3.85 ± 0.93^aB^	0.09 ± 0.00^bB^	318.34 ± 4.92^aA^	5.68 ± 1.23^aA^	0.12 ± 0.00^aA^	322.88^a^	5.00^b^	0.11^b^	188.32^a^	4.34^a^	0.10^a^
42	42.00 (+1)	0.50 (−1)	25.00 (−1)	195.00 (+1)	42.00 (+1)	205.66 ± 10.26^aB^	5.37 ± 1.27^aA^	0.14 ± 0.00^aA^	337.53 ± 2.45^aA^	5.96 ± 0.39^aA^	0.08 ± 0.00^bB^	321.36^b^	5.18^b^	0.09^a^	210.84^a^	5.20^b^	0.12^b^
43	18.00 (−1)	1.20 (+1)	41.00 (+1)	195.00 (+1)	42.00 (+1)	215.85 ± 15.49^aB^	4.12 ± 0.03^bB^	0.11 ± 0.00^aB^	482.21 ± 7.10^aA^	8.99 ± 1.43^aA^	0.12 ± 0.00^bA^	409.55^b^	7.49^b^	0.13^a^	210.06^b^	4.54^a^	0.10^b^
44	18.00 (−1)	0.50 (−1)	41.00 (+1)	80.00 (−1)	42.00 (+1)	174.20 ± 18.87^aB^	3.02 ± 0.64^aB^	0.04 ± 0.00^aA^	307.01 ± 4.58^aA^	3.94 ± 1.43^aA^	0.04 ± 0.00^aA^	278.76^b^	3.24^b^	0.03^b^	157.59^b^	3.21^a^	0.03^b^
45	30.00 (0)	0.85 (0)	33.00 (0)	137.50 (0)	29.75 (0)	207.86 ± 11.69^aB^	4.44 ± 1.96^aA^	0.10 ± 0.00^aB^	318.34 ± 4.96^aA^	5.14 ± 0.45^aA^	0.11 ± 0.00^aA^	322.88^a^	5.00^a^	0.11^a^	188.32^b^	4.34^b^	0.10^a^
46	18.00 (−1)	0.50 (−1)	25.00 (−1)	195.00 (+1)	42.00 (+1)	218.61 ± 14.57^aB^	5.69 ± 0.40^bA^	0.09 ± 0.00^bB^	343.48 ± 12.00^aA^	4.22 ± 0.00^bB^	0.11 ± 0.00^aA^	328.07^b^	4.64^a^	0.09^b^	183.55^b^	6.86^a^	0.10^a^
47	42.00 (+1)	0.50 (−1)	41.00 (+1)	195.00 (+1)	17.50 (−1)	202.82 ± 14.76^aB^	3.21 ± 0.16^aB^	0.12 ± 0.00^aA^	288.03 ± 4.56^aA^	7.24 ± 0.13^aA^	0.05 ± 0.00^bB^	280.42^b^	7.22^a^	0.06^a^	201.79^a^	3.26^a^	0.10^b^
48	42.00 (+1)	1.20 (+1)	41.00 (+1)	80.00 (−1)	17.50 (−1)	151.28 ± 5.61^bB^	3.02 ± 0.87^aA^	0.03 ± 0.00^aB^	304.67 ± 5.26^aA^	3.67 ± 0.58^aA^	0.09 ± 0.00^bA^	286.95^b^	2.49^b^	0.11^a^	168.16^a^	3.25^a^	0.02^b^
49	42.00 (+1)	1.20 (+1)	25.00 (−1)	80.00 (−1)	42.00 (+1)	142.22 ± 16.23^aB^	3.30 ± 0.02^bA^	0.08 ± 0.00^aA^	332.96 ± 12.92^aA^	3.03 ± 0.26^aA^	0.07 ± 0.00^aB^	303.11^b^	3.10^a^	0.07^a^	139.80^b^	4.43^a^	0.08^a^
50	42.00 (+1)	0.50 (−1)	41.00 (+1)	195.00 (+1)	42.00 (+1)	178.88 ± 12.68^aB^	3.26 ± 0.16^aB^	0.10 ± 0.00^aA^	272.16 ± 2.49^aA^	5.96 ± 0.71^aA^	0.02 ± 0.00^aB^	255.81^b^	5.73^a^	0.02^a^	182.05^a^	3.19^a^	0.09^b^
51	42.00 (+1)	0.50 (−1)	25.00 (−1)	80.00 (+1)	17.50 (−1)	367.95 ± 14.57^aA^	2.88 ± 0.33^bB^	0.08 ± 0.00^aB^	307.58 ± 4.24^bB^	6.33 ± 0.71^aA^	0.19 ± 0.00^aA^	314.38^a^	6.44^a^	0.16^b^	341.86^b^	3.20^a^	0.08^a^
52	18.00 (−1)	0.50 (−1)	41.00 (+1)	195.00 (+1)	17.50 (−1)	163.87 ± 11.37^aB^	4.40 ± 0.32^aB^	0.15 ± 0.00^aA^	300.64 ± 27.26^aA^	7.16 ± 0.00^aA^	0.06 ± 0.00^aB^	310.20^a^	5.68^b^	0.04^b^	145.07^b^	4.69^a^	0.14^b^
53	42.00 (+1)	1.20 (+1)	41.00 (+1)	195.00 (+1)	17.50 (−1)	166.24 ± 0.00^bB^	3.02 ± 0.75^bB^	0.06 ± 0.00^bB^	378.94 ± 2.42^aA^	7.02 ± 0.32^aA^	0.09 ± 0.00^aA^	356.82^b^	6.96^a^	0.07^b^	195.56^a^	3.84^a^	0.07^a^
54	42.00 (+1)	1.20 (+1)	25.00 (−1)	195.00 (+1)	42.00 (+1)	206.68 ± 9.31^aB^	5.68 ± 0.71^aA^	0.09 ± 0.00^aA^	364.04 ± 4.53^aA^	5.22 ± 1.04^aA^	0.04 ± 0.00^aB^	348.52^b^	5.15^a^	0.04^a^	184.67^b^	5.05^b^	0.08^b^

X_1_, soaking time (h); X_2_, salt concentration (%); X_3_, soaking temperature (°C); X_4_, germination time (h); X_5_, maturation time (h).

Values with identical upper and lower case letters on the same line do not differ significantly (*p* > 0.05).

##### 2.2.2.3. Mathematical model proposal

The polynomial model was chosen to verify the influence of randomized experiments and factors on the different responses. The proposed model is presented by Equation (1) below:


(1) $$\begin{array}{l} \text{Y}={\text{I + ax}}_{1}{\text{ + bx}}_{2}{\text{ + cx}}_{2}{\text{ + dx}}_{2}{\text{ + ex}}_{2}{\text{ + fx}}_{1}{\text{x}}_{2}{\text{ + gx}}_{1}{\text{x}}_{3}{\text{ + hx}}_{1}{\text{x}}_{4}\\ \;{\text{ + ix}}_{1}{\text{x}}_{5}{\text{ + jx}}_{2}{\text{x}}_{3}{\text{ + kx}}_{2}{\text{x}}_{4}{\text{ + lx}}_{2}{\text{x}}_{5}{\text{ + mx}}_{3}{\text{x}}_{4}{\text{ + nx}}_{3}{\text{x}}_{5}{\text{ + ox}}_{4}{\text{x}}_{5}\\ \;{\text{ + px}}_{1}^{2}{\text{ + qx}}_{2}^{2}{\text{ + rx}}_{3}^{2}{\text{ + sx}}_{4}^{2}{\text{ + tx}}_{5}^{2}\text{ + ε}(\text{error}) \end{array}$$

On the performance of the experiments, the proteolytic activity, FAN, and soluble protein content are taken as the answer (Y); I the constant; a, b, c, d, and e the linear coefficients; f, g, h, i, j, k, l, m, n, and o the interaction coefficient; and p, q, r, s, and t the square coefficients. We, therefore assumed a second-degree model for five variables.

##### 2.2.2.4. Validation of the models

To predict and accept the responses in the area defined for the study, some parameters were determined (15): the determination coefficient (*R*^2^), the absolute mean deviation analysis (AMDA), and the bias factor (Bf).

##### 2.2.2.5. Evaluation of responses

Determination of the proteolytic activity of crude extracts, free amino nitrogen, and total soluble content of the sample was performed to evaluate the influence of different factors (five factors). They were evaluated using, respectively, azocasein presented by Garcia de Fernando and Fox (16), European Brewery Convention (17) using glycine as a reference amino acid, and the biuret method with bovine serum albumin (BSA) as standard.

##### 2.2.2.6. Characterization of optimal sample

###### 2.2.2.6.1. Determination of amylolytic activity (diastatic power) and α-amylase activity

The saccharogenic method described by Bernfeld (18) was used to evaluate the diastasic power and α-Amylase activity of optimal samples of both varieties. For α-Amylase, extracts were produced with CaCl_2_ solution (3.3 g/l) and inactivation of β-amylase at 68°C for 15 min.

###### 2.2.2.6.2. Malt yield, malt loss, and Kolbach index

The method described by Embashu et al. (19) was used to determine the malt yield, malt loss, and Kolbach index following Equations (2)–(4):


(2) $$Malt\;loss\;\left(\%\right)=\frac{\text{corn weight }\left(\text{g}\right)-\text{malt weight }\left(\text{g}\right)}{\text{corn weight }\left(\text{g}\right)}*\text{100}$$

(3) Malt yield (%)=100−Malt loss (%)

(4) $$\begin{array}{l} Kolbach\;Index\;\left(\%\right)\\ =\frac{\text{soluble protein content}}{\text{crude protein content of the sample}}*\text{100} \end{array}$$

Where: n24, n48, n72 are numbers of germinated kernels at 24, 48, and 72 h.

###### 2.2.2.6.3. Physico-chemical characterization and functional properties of the *Atp-Y* and *Coc*a*-sr* optimal maize meals

The proximate composition (moisture, crude protein, crude fat, crude fiber, total digestible carbohydrate, and energy calorie) analyses of different samples were done in triplicates according to standard methods described by AOAC (20). Crude lipids were extracted in Soxhlet and protein determination was done by adopting the Kjeldahl method. Reducing sugar was evaluated through Fischer and Stein's (21) method. The standard method described by AOAC (20) also made it possible to assess the carbohydrate content using Equation (5):


(5) $$\begin{array}{r} \text{Digestible carbohydrates content }\left(\%\right)\\ =100-(\text{moisture }\left(\%\right)+\text{ash}\left(\%\right)+\text{proteins }\left(\%\right)\\ +\text{lipids }\left(\%\right)+\text{Fibers }(\%)) \end{array}$$

The starch content was evaluated as described by Jarvis and Walker (22). The amylose content of the different samples was quantified using the rapid colorimetric method illustrated by Chrastyl (23). The amylopectin content was deducted from the amylose content, with the difference in relation to the starch content of the samples being determined by the following Equation (6) below:


(6) $$\begin{array}{l} Amylopectin\;content\;\left(\%\right)=100-amylose\;content\;\left(\%\right) \end{array}$$

The mineral content (Ca, Mg, Na, K, and Cu) was assayed through the standard method defined by AOAC (20) after incinerating the sample at 560°C in an oxidizing atmosphere and analyzed using atomic absorption spectrophotometry. The phytate content of our different samples was determined using the iron III chloride colorimetric titration method described by AOAC (20). The potassium permanganate colorimetric titration of Day and Underwood (24) was applied to quantify the oxalates. The trypsin inhibitor content of the samples was determined by the standard AOAC (20) method using albumin as a control. The trypsin inhibitory capacity was calculated from Equation (7).


(7) $$\begin{array}{l} Trypsin\;inhibitor\;(\frac{mg}{100}g)\\ \text{=}\frac{(\text{Abs Standard-Abs sample})\times \text{dilution factor}}{\text{19}\times \text{sample }(\text{g})}\times \text{100} \end{array}$$

The picrate paper methodology of Makkar et al. (25) was used to quantify the hydrocyanic acid released from the sample. For phenols and flavonoids, the protocols using the Folin–Ciocalteu reagent of Gao et al. (26) allowed their quantification. The results were expressed as mg GAE/g and mg EC/g, respectively. Condensed and hydrolyzed tannins were, respectively, determined using vanillin-HCl and ferric chloride methods as described by Gaytán-Martínez et al. (27). Saponins were quantified using the standard method described by AOAC (20). The method described by Okaka et al. (28) was used to evaluate bulk density. The following equations (Equations 8–10) were applied to calculate bulk and tap densities, Hausner ratio, and porosity.


(8) $$\text{Bulk and tapped density = }\frac{\text{weight of sample }(\text{g})}{\text{volume occupied by the sample}}$$

(9) $$Hausner\;ratio=\frac{\text{tapped density}}{\text{bulk density}}$$

(10) $$Porosity\;\left(\%\right)={\frac{\text{tapped density-bulk density}}{\text{tapped density }}}^{*}\text{100}$$

Concerning pH, it was determined using a pH meter (GlowGeek Advanced Portable pH meter) according to the standard method described by AOAC (20). The standard method described by AFNOR (29) was used to evaluate titrable acidity. For functional properties, swelling rate and water holding capacity were determined following the methods described by Okezie and Bello (30) modified by Tambo et al. (8, 9) and Lin et al. (31), respectively.

###### 2.2.2.6.4. Amino acid analysis

Extraction was done using the modified procedure of Bidlingmeyer et al. (32) previously described by Chinma et al. (33). Subsequent profiling of the AA composition of the samples was determined using a liquid chromatography-Mass spectrometer (Quasar, PerkinElmer, Hopkinton, USA) coupled with a photodiode array detector (model MD-2010; JASCO, Tokyo, Japan), operating at 254 nm. From the results obtained, the total AAs (TAAs), total EAAs (TEAAs), total NEAAs (TNEAAs), total acidic AAs (TAAAs) comprising glutamic and aspartic acids (34), total basic AAs (TBAAs) involving lysine, arginine, and histidine (35), total neutral AAs (TNAAs) calculated as TAAs – (TAAAs + TBAAs), total sulfur AAs (TSAAs) from methionine and cysteine levels (36), percentage cysteine in TSAAs, and total aromatic AAs (TArAAs) based on phenylalanine and tyrosine contents (36) were estimated. In addition, the ratio of TEAAs/TNEAAs (37, 38) and leucine to isoleucine (Leu/Ile) ratio (39) was evaluated.

#### 2.2.3. Statistical analysis

The values obtained were expressed as mean ± standard deviations using the analysis of variance (ANOVA) to determine the degree of significance (*p* < 0.05). The significance of each factor was determined by the Fisher test. The regression equations were also subjected to the Fisher test to determine the coefficient of determination *R*^2^. The calculations were carried out using MINITAB 18.0 software (IBM, USA). The accepted confidence level was *p* < 0.05. Graphical representations of the iso and surface response curves of the postulated models were made using SIGMA PLOT 12.0 software. IBM SPSS^TM^ software version 25.0 and the Duncan test allowed us to verify the existence of significant differences between the predicted and experimental responses within the two maize varieties.

## 3. Results and discussion

### 3.1. Modeling

The experimental matrix present in Table 1 was used to set up the mathematical model translating the influence of the five factors (soaking time, germination time, maturation time, plant salt concentration, and soaking temperature) chosen on the responses (Y) studied. It was found that variety and malting conditions significantly (*p* < 0.05) influence the different responses. Free amino nitrogen content in the malt is a determining factor for malt quality. These compounds are metabolized into ketone and aldehyde derivatives, which are responsible for the flavor of the beer by the microorganisms during fermentation. They are also a source of energy for the microorganisms. The evaluation of the free amino acid (FAN) content shows that it varies from 72.93 mg/100 g (trial 8) to 431.55 mg/100 g (trial 1) for the *Atp-Y* variety and from 185.99 mg/100 g (trial 22) to 482.21 mg/100 g (trial 43) for the *Coca-sr* variety. Furthermore, the experimental and predicted values of the model of both varieties presented perfect matches in the different tests; proof of the quality of the manipulations and the methodology used. In general, the *Coca-sr* variety presented the highest values of free amino acids, which could be explained by the increased synthesis of protein-digesting enzymes such as aminopeptidases, carboxypeptidases, peptidases, and endoproteases of all kinds in this variety, which are responsible for protein digestion (40). This would also be due to the high content of soluble proteins which are mostly enzymes. Indeed, Tambo et al. (41) showed that there is a positive and significant correlation between soluble protein content and enzyme activity. The values obtained in both varieties are significantly (*p* < *0.05*) higher than those of Embashu et al. (19) which were 111.80 and 167.20 mg/100 g dry matter in two sorghum varieties, respectively. These results further show that these two maize varieties would be better suited for the substitution of sprouted barley in beer production as well as malt beverages.

The evaluation of soluble protein content showed that it varied from 1.92 mg (trial 8) to 9.35 mg (trial 10) and from 2.20 (trial 29) to 8.43 (trial 43) for the varieties *Atp-Y* and *Coca-sr*, respectively. The experimental and calculated values of the two varieties were almost all non-significantly different. In general, *Coca-sr* had the highest soluble protein content. This could be explained by a strong activation of hormones (gibberellins) responsible for the lifting of the dormant state of the catabolic enzymes of the seed. In addition, it would also be due to an increased release of soluble proteins and peptides following the dissociation of complexes formed between them and other macromolecules. Indeed, Narsih et al. (42) demonstrated that during germination, insoluble organic complexes were broken down by hydrolytic enzymes neo-synthesized into soluble low molecular weight organic molecules.

The evaluation of the proteolytic activity in these two varieties shows that it is very low and that cereals are not a priority source of proteases. It varies from 0.03 IU (trial 48) to 0.15 IU (trial 52) and from 0.02 IU (trial 14) to 0.19 IU (trial 51) for the varieties *Atp-Y* and *Coca-sr*, respectively. This parameter is significantly affected by the variety as well as the production conditions. Indeed, the *Coca-sr* variety presented the highest proteolytic activities as a consequence of a strong synthesis of proteolytic enzymes in these grains. This would be linked to better extraction of proteolytic enzymes from this variety. Colored cereals are richer in lipids than uncolored cereals. These lipids form complexes with these proteins in the cereal membranes, making them insoluble and difficult to extract. Moreover, these lipids confer a certain membrane rigidity to these cereals thus limiting the extraction of soluble compounds like proteins.

### 3.2. Analysis of variance and validation of mathematical models

Table 2 below presents the validation of the mathematical models, contribution of variables, coefficient, *P*-value, and sums of squares of the variables. The table shows that the factors and their effects were significant in all mathematical models of the two varieties. This also demonstrates that these mathematical models could be applied in the production of malts with free amino acid contents which are of crucial importance during fermentation in the brewing industry. Table 2 also shows that the FAN (free amino acid) content of *Coca-sr* is significantly (*p* < *0.05*) and negatively (see coefficient of variables in Table 2) affected by the quadratic effects of germination and maturation time, soaking time–soaking temperature, soaking time–sprouting time, and soaking temperature–maturing time interactions. On the other hand, it is positively affected by the linear effects of the germination and ripening time, the quadratic effect of the soaking time, and the interactions input concentration–soaking temperature and soaking temperature–germination time.

**Table 2:** Validation of mathematical models, contribution of variables, coefficient, *P*-value, and sums of squares of the variables.

	***Coca-sr***												***Atp-Y***											
	**FAN**				**Soluble protein**				**Proteolytic activity**				**FAN**				**Soluble protein**				**Proteolytic activity**			
**Source**	**Coeff**	**RC (%)**	***P***	**SS**	***P***	**RC (%)**	**SS**	**Coeff**	***P***	**RC (%)**	**SS**	**Coeff**	***P***	**RC (%)**	**SS**	**Coeff**	***P***	**RC (%)**	**SS**	**Coeff**	***P***	**RC (%)**	**SS**	**Coeff**
Model		82.99	**0.000**	94,188	**0.000**	83.98	88.678		**0.000**	85.51	0.052229		**0.000**	90.53	160,778		**0.000**	84.69	90.747		**0.000**	86.00	0.0410	
X_i_ (linear)	322.88	31.96	**0.000**	36,273	**0.000**	48.53	51.246	4.996	**0.000**	14.16	0.008652	0.11014	**0.000**	27.81	49,395	188.32	**0.000**	34.28	36.737	4.344	**0.000**	45.86	0.0219	0.09841
X_1_	8.17	0.45	0.355	515	0.628	0.12	0.123	0.126	0.849	0.02	0.000010	−0.00113	0.075	0.97	1,722	14.94	**0.003**	4.78	5.127	−0,815	**0.012**	2.99	0.0014	−0.01360
X_2_	17.17	2.00	0.057	2,274	0.097	1.42	1.499	−0.441	**0.032**	2.20	0.001342	0.01319	**0.000**	5.17	9,175	−34.48	0.765	0.04	0.045	−0.077	**0.001**	5.39	0.0026	−0.01826
X_3_	14.81	1.49	0.098	1,692	0.426	0.32	0.333	−0.208	**0.002**	4.92	0.003005	−0.01973	**0.000**	12.01	21,331	−52.58	**0.000**	11.67	12.501	−1.273	**0.003**	4.53	0.0022	−0.01673
X_4_	53.85	19.72	**0.000**	22,377	**0.000**	45.11	47.638	2.485	**0.006**	3.86	0.002360	−0.01749	0.374	0.23	414	−7.32	**0.000**	14.10	15.104	1.399	**0.000**	32.56	0.0155	0.04485
X_5_	34.93	8.30	**0.000**	9,415	0.082	1.57	1.653	−0.463	**0.011**	3.17	0.001935	−0.01584	**0.000**	9.43	16,753	−46.59	**0.008**	3.70	3.960	0.716	0.346	0.39	0.0002	−0.00489
X_i_X_i_ (square)		18.78	**0.000**	21,317	0.248	3.41	3.606		**0.000**	27.13	0.016569		**0.000**	36.65	65,082		**0.000**	33.98	36.414		**0.000**	15.35	0.0073	
X_1_X_1_	43.00	4.22	**0.020**	4,793	**0.027**	2.71	2.857	−1.198	0.558	0.00	0.000000	−0.0070	0.139	0.88	1,557	−24.80	**0.003**	5.10	5.467	−1.653	0.642	0.01	0.0000	−0.0048
X_2_X_2_	−13.90	0.09	0.432	102	0.579	0.14	0.151	0.291	**0.000**	6.87	0.004194	−0.0520	0.271	0.25	447	18.30	0.369	0.36	0.388	0.466	**0.002**	4.38	0.0021	−0.0353
X_3_X_3_	−18.60	0.26	0.297	299	0.504	0.26	0.273	−0.351	**0.000**	17.66	0.010788	−0.0770	**0.000**	6.23	11,059	−69.70	**0.004**	5.11	5.475	−1.602	**0.000**	8.51	0.0041	−0.0463
X_4_X_4_	−53.70	4.18	**0.004**	4,747	0.676	0.07	0.078	0.219	0.186	0.69	0.000419	−0.0160	0.150	1.10	1,955	−24.10	**0.000**	20.18	21.624	3.302	0.063	1.68	0.0008	−0.0198
X_5_X_5_	−77.30	10.02	**0.000**	11,375	0.492	0.23	0.247	0.361	**0.045**	1.91	0.001168	−0.0248	**0.000**	28.19	50,065	162.20	**0.013**	3.23	3.459	−1.348	0.186	0.77	0.0004	0.0139
X_i_X_j_ (interactions)		32.25	**0.000**	36,598	**0.000**	32.03	33.827		**0.000**	44.22	0.027008		**0.000**	26.07	46,300		**0.003**	16.42	17.596		**0.000**	24.80	0.0118	
X_1_X_2_	12.10	0.13	0.616	150	0.291	0.56	0.590	−0.760	**0.000**	9.00	0.005499	−0.0734	**0.000**	7.08	12,568	−110.90	0.619	0.12	0.125	−0.351	**0.013**	2.90	0.0014	−0.0368
X_1_X_3_	−67.60	4.11	**0.008**	4,666	0.554	0.17	0.183	−0.424	**0.015**	2.89	0.001763	−0.0415	0.976	0.00	0	0.70	0.238	0.67	0.719	0.839	0.071	1.47	0.0007	−0.0262
X_1_X_4_	−58.30	3.06	**0.020**	3,469	**0.013**	3.36	3.551	1.865	**0.007**	3.66	0.002236	−0.0468	0.208	0.47	841	−28.70	**0.000**	7.17	7.687	−2.744	0.257	0.56	0.0003	−0.0162
X_1_X_5_	−35.30	1.12	0.150	1,273	**0.015**	3.21	3.386	−1.821	**0.000**	7.64	0.004668	−0.0676	0.079	0.94	1,674	−40.50	0.466	0.25	0.270	0.514	**0.000**	7.70	0.0037	0.0600
X_2_X_3_	112.30	11.34	**0.000**	12,871	**0.006**	4.19	4.424	2.082	**0.000**	19.57	0.011954	0.1082	0.866	0.01	15	3.80	0.706	0.07	0.072	0.265	**0.007**	3.52	0.0017	0.0406
X_2_X_4_	92.20	7.65	**0.001**	8,681	0.058	1.87	1.977	1.391	0.885	0.01	0.000006	−0.0024	**0.000**	12.45	22,117	147.20	0.119	1.19	1.277	1.11	0.051	1.75	0.0008	−0.0285
X_2_X_5_	43.40	1.69	0.079	1,920	**0.002**	5.63	5.948	2.414	0.363	0.37	0.000228	0.0150	0.288	0.33	594	−24.10	0.287	0.54	0.583	−0.756	**0.039**	1.97	0.0009	0.0303
X_3_X_4_	15.20	0.21	0.529	237	**0.000**	12.02	12.689	3.525	0.415	0.30	0.000183	0.0134	**0.029**	1.50	2,658	51.00	0.149	1.01	1.083	−1.030	**0.026**	2.30	0.0011	0.0327
X_3_X_5_	−53.90	2.61	**0.031**	2,962	0.160	1.00	1.058	1.018	0.619	0.11	0.000068	−0.0081	0.855	0.01	17	−4.10	**0.004**	4.45	4.772	−2.162	0.719	0.06	0.0000	−0.0051
X_4_X_5_	−19.00	0.32	0.433	368	0.846	0.02	0.020	0.139	0.229	0.66	0.000403	0.0199	**0.002**	3.27	5,815	75.50	0.164	0.94	1.008	−0.993	**0.019**	2.58	0.0012	−0.0347
Error		17.01		19,301		16.02	16.922			14.49	0.008851			9.47	16,813			15.31	16.410			14.00	0.0067	
Inadequation		16.57	**0.000**	18,799	0.120	12.80	13.512		**0.000**	14.27	0.008715		**0.006**	8.52	15,137		**0.009**	13.69	14.669		**0.036**	11.94	0.0057	
Pure error		0.44		501		3.23	3.410			0.22	0.000136			0.94	1,677			1.62	1.740			2.06	0.0010	
		100.00				100.00				100.00				100.00				100.00				100.00		
Validation of the model	107,156												100,00%											
*R* ^2^		82.99				83.98			85.51	90.53				84.69				86.00						
Adjust *R*^2^		72.69				74.26			76.73	84.79				75.41				77.52						
Bias factor		1.002				0.998			1.002	1.02				1.01				0.98						
AMDA		**0.24**				**0.008**			**0.02**	**0.03**				**0.10**				0.03						

Coeff, coefficient value; RC, real contribution; SS, sum of square; p, *P*-value; AMDA, absolute mean deviation analysis; *R*^2^, determination coefficient; FAN, free amino nitrogen.

X_1_, soaking time (h); X_2_, salt concentration (%); X_3_, soaking temperature (°C); X_4_, germination time (h); X_5_, maturation time (h). The bold values represent those that significantly influence the responses.

The FAN content of the *Atp-Y* variety is significantly (*p* < *0.05*) and negatively affected by the linear effects of input concentration, soaking temperature, and ripening time; the quadratic effect of soaking temperature and the interaction between soaking time and soaking temperature. On the other hand, a positive effect of the soaking time, the quadratic effect of the ripening time, the interactions between input concentration and germination time, soaking temperature and germination time, and finally germination time and ripening time are observed. The observed difference in the action of the factors as well as their direction of variation on the two varieties would be the effect of the pectocellulosic and nutritional composition of the two varieties (43). It follows that a 2-fold increase in germination and maturation times for the *Coca-sr* variety would result in the use of free amino acids from catabolism for the production of energy needed for anabolism. It would also be linked to the mobilization of free amino groups in metabolic processes as well as their use as precursors in the synthesis of soluble proteins (44, 45). The combination of soaking and temperature leads to an embrittlement of the cellulosic membrane leading to a loss of free amino acids in the soaking water by leaching due to their soluble character. In addition, an increase in the soaking time associated with germination would lead to a dilution of the reagents due to the high water content of the seed and consequently a low proteolytic activity during germination (46). It would also be linked to an inhibition of enzyme activity. The positive effect of germination and ripening time in this variety would be linked to the activation of protein hydrolytic enzymes such as aminopeptidases, carboxypeptidases, and endopeptidases during these two processes. Hydrolytic enzymes such as proteases require water for their action, especially for the mobility of the reagents and enzyme–substrate contact. A doubling of the soaking time, therefore, favors this action. Enzymatic activity can also be favored by certain ions (calcium, sodium, potassium, iron, etc.) provided by the plant input used during soaking, and their stability is ensured by these at certain temperatures (41). Similarly, the activation energy of the enzymes would be lowered at optimal temperature. This would explain an improvement of the FAN content with the combinations of input concentration–soaking temperature and soaking temperature–germination time. In addition to the activating ions, the plant input would also provide ions that could inhibit the activity of the enzymes present in the *Atp-Y* variety, which would reflect the negative effect of this factor on the FAN content. The drop in hydrolytic activity of proteases due to the loss of the three-dimensional configuration (breaking of low-energy bonds such as ionic and hydrogen bonds stabilizing this structure) of the enzymes with increasing temperature would also explain the lowering of the FAN content with increasing temperature (47, 48). Tambo et al. (8, 9, 41) also reported that although enzyme activity changed with temperature, it was also lowered with a large temperature increase.

As for the soluble protein content, it is significantly (*p* < *0.05*) lowered by the quadratic effect of soaking time and soaking time–maturing time interaction for the *Coca-sr* variety. The *Atp-Y* variety, on the other hand, is lowered by the linear effects of soaking time and soaking temperature, their quadratic effects as well as that of ripening time, and the soaking time–sprouting time and soaking temperature–ripening time interactions. There is a dilution and leaching of soluble proteins leading to a loss of those of the seed with the increase of the soaking time and the embrittlement of the cellulosic wall with the increase of the temperature. It would also be linked to the destruction of their structure, notably the breaking of peptide bonds under the effect of temperature (49). Tsopbeng et al. (12) and Klang et al. (50, 51) also reported that the content of synthesized proteins strongly depended on the soaking time, and therefore on the water content, germination, and maturation time. Indeed, these authors reported that above a certain water content, there was an inhibition of the action of gibberellins responsible for the lifting of the dormancy state of soluble proteins, which are endogenous enzymes, as well as a reduction in their content including enzyme activities beyond certain periods of germination and maturation. This response for the *Coca-sr* variety was nevertheless improved (*p* < *0.05*) by the linear effect of germination time, soaking time–germination time, input concentration–soaking temperature, input concentration–maturing time, and soaking temperature–maturing time interactions. The positive and significant (*p* < *0.05*) effect of germination and ripening time as well as the quadratic effect of germination time was observed in the *Atp-Y* variety. This is explained by the dislocation of insoluble complexes (phenols-protein, lipid-protein, tannin-protein...) by enzymes neo-synthesized during germination and ripening leading to the release of soluble proteins (42). Gujjaiah and Kumari (45) and Rodríguez et al. (52) and also reported an improvement in the content of protein-like soluble organic molecules with the germination process as a result of the mobilization of reserve amines from the cotyledons and the FANs released for their synthesis.

The effect of variables on proteolytic activity was also assessed. *Coca-sr* was negatively affected by linear effects of soaking temperature, germination time, and ripening time; quadratic effects of plant salt concentration, soaking temperature, and ripening time; interactions between soaking time and all other variables. The proteolytic activity in the *Atp-Y* variety was lowered by the linear effects of the first three variables, the quadratic effect of variables 2 and 3, the soaking time–input concentration interaction, and germination–maturation time interaction. Indeed, there is a loss of protease conformation under the effect of temperature and thus a reduction in their digestive capacity. Moreover, inhibition of the proteolytic activity by the metal ions contained in the soaking salt would also be at the origin of the decrease of this activity by this variable within the *Atp-Y* variety. This activity is on the other hand significantly improved by the concentration of vegetable salt and the interaction between the concentration of vegetable salt and the soaking temperature for the *Coca-sr* variety. A positive effect was observed with increasing germination time, soaking time–maturing time, plant salt concentration–soaking temperature, salt concentration–maturing time, and soaking temperature–germination time interactions for the *Atp-Y* variety. This difference observed between the two varieties in terms of the variables activating and inhibiting their activities would be related to a difference in the mechanism of action of the enzymes of the different varieties and a different composition of amino acids at the active site catalyzing these reactions. It is also evidence that the composition and class of enzymes are influenced by variety. Laetitia et al. (40) reported an improvement in the digestive capacity of cereals after soaking and germination. According to the same authors, this could also be explained by a reduction in the content of anti-nutrients such as phytates, which can complex the cations that activate enzyme activity, thus preventing them from acting as enzyme cofactors.

Table 2 also presents the sum of squares and the contribution of the different variables. These two parameters are significantly (*p* < *0.05*) correlated with the *p*-value and the coefficient of the variables. They also confirm the degree of significant influence of each factor. It follows that all the factors that significantly influenced the responses of the two varieties presented high coefficients and sums of squares according to the degree of influence of the factor. The interactive, linear, and interactive effects contributed most to the FAN, soluble protein, and proteolytic activity content, respectively, for the *Coca-sr* variety. On the other hand, the quadratic effects of ripening and germination times and the linear effect of the latter contributed most to the different responses (FAN content, soluble protein content, and proteolytic activity, respectively) in the *Atp-Y* variety.

Some parameters such as determination coefficient (*R*^2^), the adjusted coefficient of determination (*R*^2^ adj), the absolute average deviation analysis (AADM), and the bias factor (bf) are used to check the robustness of the model, the adequacy between the predicted and experimental models, and the degree of concordance between the selected variables and the evaluated responses (43, 53). The *R*^2^ and adj *R*^2^ ranged from 82.99 to 90.53% and 72.69 to 84.79%, respectively. These two parameters are positively correlated and have all presented values above the standard values (*R*^2^ and *R*^2^ adj ≥ 75%) for validation of a mathematical model (54, 55). It can also be seen from Table 2 below that the absolute average deviation analysis (AADM) and the bias factor (bf) have values within the range of standard values (0–0.30 for AADM and 0.75–1.25 for bf) for all the responses evaluated regardless of the variety (56, 57).

### 3.3. Effect of different factors on responses

#### 3.3.1. Effect of soaking time

**Figure 2:**
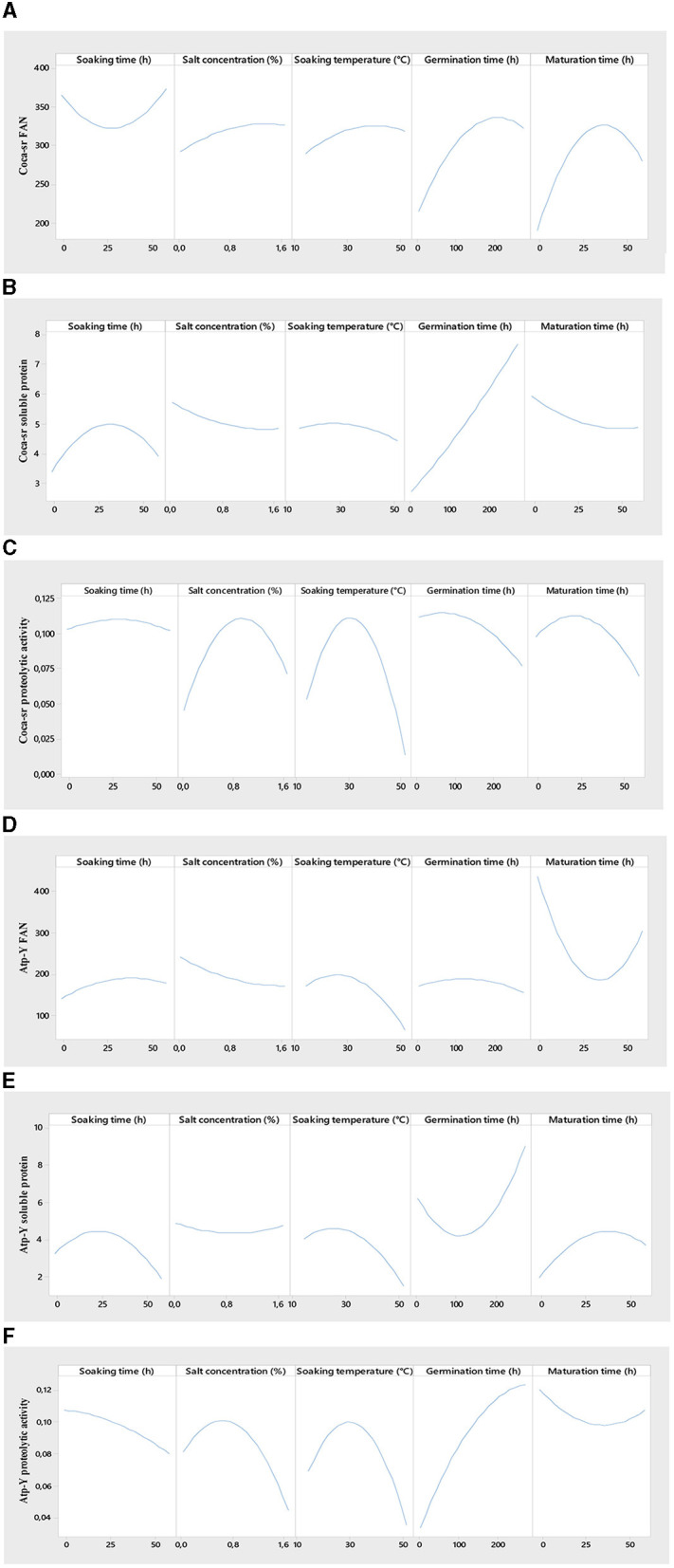
Effect of individual variables on free amino acid content [**(A, D)** for *Coca-sr* and *Atp-Y*, respectively], soluble protein content [**(B, E)** for *Coca-sr* and *Atp-Y*, respectively], and proteolytic activity [**(C, F)** for *Coca-sr* and *Atp-Y*, respectively].

Figure 2 shows the effect of individual variables on the evaluated responses. Steeping plays a major role in the malting process. It represents the first step in the activation process of gibberellins hormones responsible for lifting. It showed an overall positive effect on the soluble protein content of both varieties, the proteolytic activity of the *Coca-sr* variety, and the FAN content of the *Atp-Y* variety. These parameters increase with time to an optimal value around 25 h of soaking followed by a non-significant effect on the different responses before a decrease in these. Klang et al. (43) also reported that the amylolytic activity of the paddy rice varieties *Nerica 3* and *Nerica L56* was highest between 20 and 25 h soaking. They attributed this to an activation of the reserve enzymes and the facilitation of their mobility following the absorption of water by the grains. It would also be the consequence of the activation of certain anti-nutrient digestion enzymes with the capacity to dissociate the insoluble complexes formed between proteins and certain compounds (phenols, tannins, phytates, etc.), thus leading to an increase in the soluble protein and FAN content (58). The drop observed beyond the peak of the various responses is the consequence of high water absorption by the seed leading to asphixis as a consequence of a reduction of the respiratory metabolic phenomena (59). A prolonged soaking time would also cause the leaching of soluble substances such as amino acids and soluble proteins, a dilution of reagents, and acidification of the medium following the fermentation of proteins and amino acids released (58, 60). In addition, an overall negative effect of steeping time on the FAN content of the *Coca-sr* variety and the proteolytic activity of the *Atp-Y* variety was observed similar to the study done by Tambo et al. (41) and Klang et al. (50, 51). Indeed, a genetic difference, the localization of proteases, and the water content would explain the drop in FAN of the *Coca-sr* variety at the beginning of steeping and the proteolytic activity of the *Atp-Y* variety throughout steeping.

#### 3.3.2. Effect of plant salt concentration

Hydrolytic enzymes are mostly metalloenzymes that require metal cofactors for their activity (41). These ions are involved in active site stabilization, binding, catalysis, and substrate transformation. This influence on catalytic activity is concentration dependent (61). It follows from Figure 2 below that the FAN content of the *Coca-sr* variety increases gradually with salt concentration until it stabilizes at 1.6%, while that of the *Atp-Y* variety decreases with time. This could be explained by the different mechanisms of action of the proteases of the two varieties, the strong presence of inhibitors (Fe^2+^, Hg^2+^, Cu^2+^, and al^3+^) of *Atp-Y* proteases in these salts as well as the compact molecular structure formed between proteins and polysaccharides at the level of the external layer of its wall (62). Furthermore, the removal of FANs by osmosis as a consequence of wall embrittlement would also explain this. The presence of activators of proteolytic activity would explain the gradual increase in the FAN content of the *Coca-sr* variety. The soluble protein content of both varieties was almost not influenced by the salt content. The proteolytic activity of both varieties gradually increased with the salt concentration until it peaked at 0.8% and then dropped. This growth can be explained by the stabilization of the conformation of the active site following the formation of electrostatic interactions between the activating cations of the proteolytic activity (Ca^2+^ and Mg^2+^) and the electronegative amino acids (aspartic and glutamic acids) as well as activation by the latter of the functional groups of the amino acids of this same site, thus favoring catalysis (12, 61). Above the threshold activator concentration, an increase in ion concentration leads to steric hindrances at the active site limiting enzyme-substrate interactions and thus the decrease in enzyme activity. Tambo et al. (41) reported an inhibitory effect of Na^+^ on the amylases of two maize varieties above 1 mM.

#### 3.3.3. Effect of soaking temperature

Temperature plays a role in the activation and formation of binding energies. In the context of this study, it favors the lifting of the dormant state of the grains by facilitating the absorption of water in particular (43). The responses gradually evolve with the soaking temperature to maxima before lowering. The high threshold temperatures were observed with the *Coca-sr* variety. The stiffer pectocellulosic wall and the endospermic localization of the proteases would explain the values for the latter. The positive evolution of the FAN content can be explained by the hydrolysis of soluble non-enzymatic proteins decomplexed from antinutrients. Indeed, the increase in temperature leads to the elimination of certain heat-sensitive antinutrients that facilitate protein digestibility (2, 63). The lowering of the FAN content beyond the optimal temperature would be linked to an increased entry of quenching water following the embrittlement of the wall leading to its asphixis. It is also the phenomenon of a progressive denaturation of enzymes (48, 49). The elimination of soluble proteins by the phenomenon of solubilization explains the drop in soluble protein content.

#### 3.3.4. Effect of germination time

Germination is the limiting stage of the malting process. During germination, the aleurone layer enzymes are activated, reserve substances are broken down, and the nutritional value of the grain is improved (43, 58). FAN content and proteolytic activity increased proportionally with germination time in both varieties. FAN maxima are reached around 200 and 150 h for *Coca-sr* and *Atp-Y*, respectively. These peaks are followed by a drop. This could be explained by a mobilization of the reserve enzymes of the endosperm and episperm in the degradation of proteins into peptides and amino acids (64). Gujjaiah and Kumari (45) also reported an improvement in soluble protein content, proteolytic activity, and protein digestion products after 192 h of germination. The observed inflection is thought to be related to the inhibition of proteolytic activity by the products of enzymatic hydrolysis, the depletion of reserve oxygen required for catabolism, the overexpression of gibberellins which has an antagonistic effect at 30 mg/L, and the use of previously released amino acids as a precursor for the synthesis of new proteins at the level of the developing embryo (64). Furthermore, a reduction in soluble protein content at the beginning of germination was observed with the *Atp-Y* variety in contrast to the *Coca-sr* variety. Senhofa et al. (64) reported that hydrolysis of reserve proteins to release substrates and energy for embryo development would explain this. Activation of phytases and other anti-nutrient degrading enzymes leads to the release of soluble compounds including proteins (62). Inyang and Zakari (65) also reported an increase in protein content in sprouted cereals and vegetables. It is also noted that a negative correlation was observed between proteolytic activity, FAN content, and soluble protein content in the *Atp-Y* variety. Genetic variability between the two maize varieties would also be the reason for this observation.

#### 3.3.5. Effect of ripening time

Maturation marks the transitional stage of malting. It is located at the gateway to anabolism and is responsible for the mobilization of all reserve enzymes activated during the previous stages. It is also the stage of overexpression of enzymes and almost complete degradation of all reserve substances such as proteins, carbohydrates, and lipids (43). It is shorter than germination (between 6 and 48 h) and does not require imbibition. Maturation showed opposite effects in both varieties. There was an increase in FAN content and proteolytic activity concomitant with a drastic drop in soluble protein content in the *Coca-sr* variety. This could be explained by the activation of exogenous proteases and the degradation of proteins in the epidermal layer (64). The drop in proteolytic activity and FAN content follows the inhibition of catabolic processes and the activation of anabolism. The *Atp-Y* variety, on the other hand, showed the opposite development of *Coca-sr*. The complete mobilization of reserve proteins during the previous stages and the use of the products of their hydrolysis for the *de novo* synthesis of these same compounds would explain this phenomenon. It would also be linked to an absence of exospermic proteases. These results also suggest that the *Atp-Y* variety requires shorter maturation times during the malting process.

### 3.4. Contour plot

#### 3.4.1. Contour plot of the trade-offs of the *Atp-Y* variety

**Figure 3:**
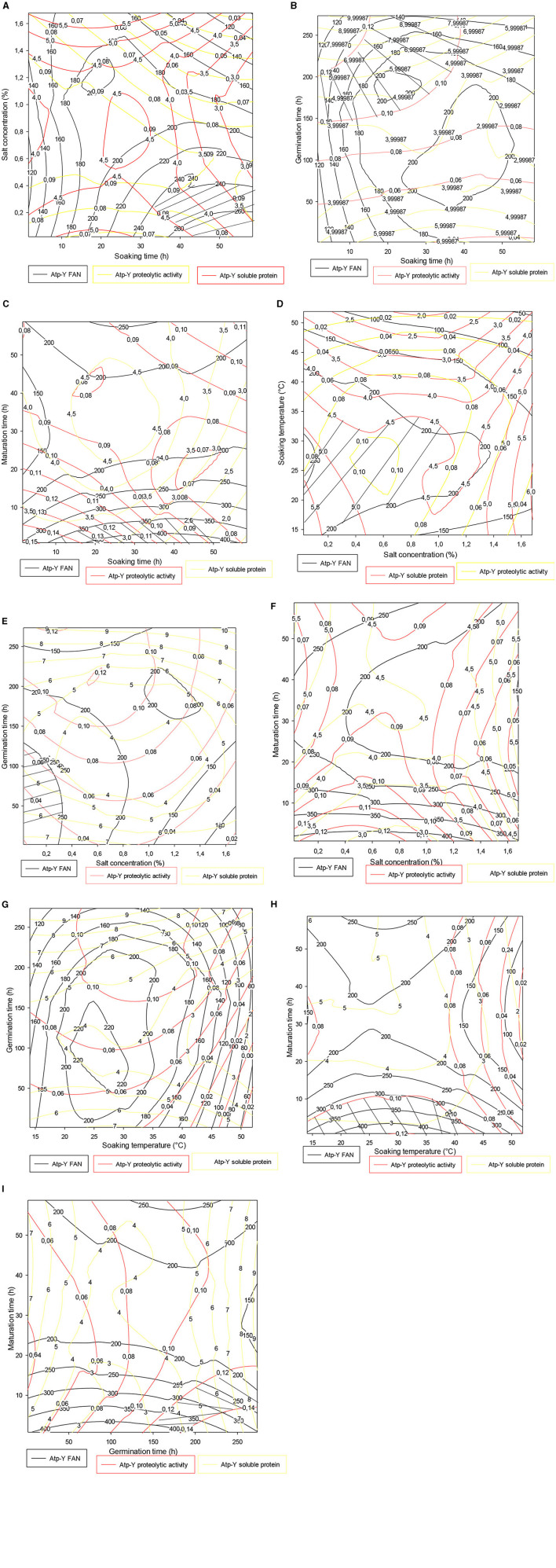
**(A–I)** Contour plot showing the trade-off areas of significant interactions on the different responses (*Atp-Y*).

Figure 3 shows the trade-off zones of the responses according to the interactions that significantly influenced the responses within the *Atp-Y* variety. The soaking time–salt concentration interaction (Figure 3A) showed an optimal zone around 50 h of soaking and 0.4% of salt concentration. The response values obtained confirmed the previous results that the use of vegetable ash combined with a long soaking time reduces the enzymatic activity of the germinating cereals. The soaking time–germination time interaction in Figure 3B shows an optimal zone centered on the germination time. This shows that a short soaking time requires a long germination time for the mobilization of reserve enzymes. A similar phenomenon was observed between soaking and ripening (Figure 3C). Figure 3D shows that the trade-off zone of the interaction between plantain peel ash concentration and the soaking temperature has respective FAN values, soluble protein, and proteolytic activity of 200 mg/100 g, 4.50 mg, and 0.09 IU, respectively. These values are lower than in Figure 3C. Indeed, the increased fragility of the membrane under the combined effect of temperature and salts leads to a reduction of metabolic phenomena due to the asphixis of the grains. Control of temperature and salt concentration during the germination process of cereals is necessary. Figures 3E, F show that the addition of 0.25% associated with 200 h of germination and between 0 and 1.40% of vegetable salt for <10 h of maturation allowed us to obtain optimal values of FAN, soluble proteins, and proteolytic activity. Figure 3G shows that soaking maize kernels of the *Atp-Y* variety at a temperature of 20–35°C followed by germination for up to 200 h yields high values of FAN, soluble protein, and proteolytic activity. Figure 3H shows an evolution of the three responses in proportion to the soaking temperature and inversely to the maturation time. Figure 3I shows that the peak of the optimal values was observed around 250 h of germination and 20 h of maturation. These results suggest that the parameters influencing the different factors should be considered when setting up a malting process with this maize variety.

#### 3.4.2. Trade-off contour plot for the *Coca-sr* variety

**Figure 4:**
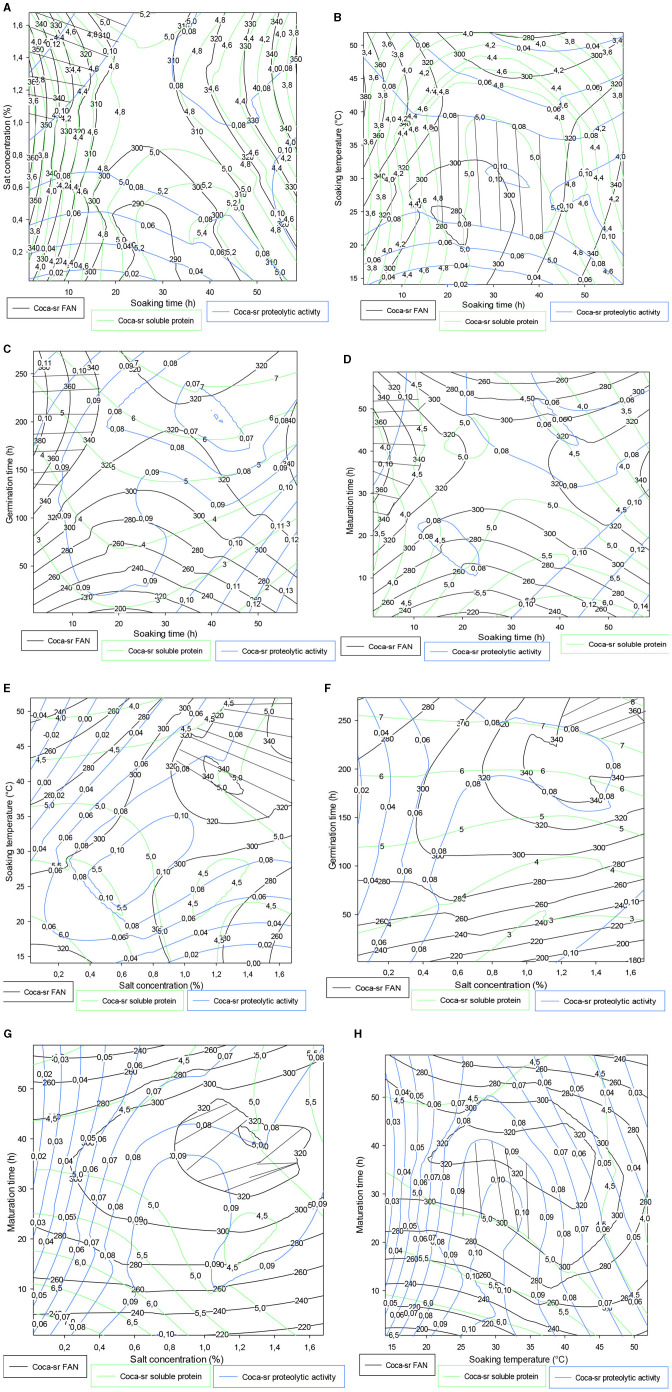
**(A–H)** Contour plot showing the trade-off areas of significant interactions on the different responses (*Coca-sr*).

It follows from Figure 4A that the FAN content, soluble protein value, and proteolytic activity gradually increase with an ash concentration in contrast to soaking time. Optimal values were obtained within 20 h of soaking and between 0.8 and 1.6% of vegetable salt concentration. Tambo et al. (13) also reported the same observations during the optimization of the amylolytic activity of these two varieties. Regarding the association of soaking time and soaking temperature, it appears that between 20 and 40 h of soaking at 20–45°C, the *Coca-sr* variety would be very apt to produce proteases capable of degrading the reserve proteins into FAN. It should be noted that the values drop drastically on either side of these optimal zones. The drop observed after these intervals can be due to losses of FAN in their degradation (13). Figures 4C, D show, respectively, that short soaking times must be associated with very long germination and ripening periods. Indeed, low seed hydration would limit the rapid lifting of the enzyme dormancy state of the seed, which would only be effective with increasing germination or ripening time. Figure 4E below shows that the compact structure of the epidermis of *Coca-sr* maize grains requires soaking temperatures close to 50°C for salt concentrations of around 2% to obtain the good hygroscopy of the seed necessary for its metabolic activities during germination. The observations made in Figure 4B were also noted in Figures 4G, H (salt concentration-maturation time and soaking temperature-maturation time combinations, respectively). Figure 4F shows that high salt concentrations (more than 1.6%) associated with very long germination times (more than 250 h) are necessary to obtain high proteolytic activity, FAN, and soluble protein contents. This would be linked to the presence of large quantities of metalloenzymes in this variety, unlike the first (*Atp-Y*).

### 3.5. Validation of optimal conditions

The predicted and experimental values of optimal conditions defined by the software are present in Table 3. These conditions allowed us to obtain optimal proteolytic activities, and high FAN and soluble protein contents. These manipulations show that the *Atp-Y* variety needs 7.31 h of soaking time in combination with 1.678% of vegetable salt at a temperature of 34.65°C followed by 245.59 h of germination time and 0.765 h of maturation time. *Coca-sr* variety requires 1.608 h of soaking time in combination with 1.678% of vegetable salt at a temperature of 51.93°C followed by 273.94 h of germination time and maturation time of 58.73 h. Klang et al. (43) also reported a variation in the optimal sprouting conditions of *Nerica 3* and *Nerica L56* rice seeds. They attributed this to a difference in epidermal composition, genetic variability, and a difference in enzyme localization zone in the two varieties (62).

**Table 3:** Validation test of optimal conditions.

**Samples**	***Atp-Y***	***Coca-sr***
**Optimal conditions**		
Soaking time (h)	7.31	1.608
Soaking temperature (°C)	34.65	51.93
Salt concentration (%)	1.678	1.678
Germination time (h)	245.59	273.94
Maturation time (h)	0.765	58.73
**Free amino nitrogen**		
Desirability	0.87	1
Experimental optimal value	483.19 ± 3.73^aB^	659.57 ± 14.28^aA^
Predicted optimal value	486.81^aB^	654.27^aA^
**Soluble protein**		
Desirability	1	1
Experimental optimal value	10.50 ± 0.12^aB^	16.79 ± 0.13^aA^
Predicted optimal value	10.28^aB^	17.27^aA^
**Proteolytic activity**		
Desirability	1	1
Experimental optimal value	0.189 ± 0.00^aB^	0.255 ± 0.00^aA^
Predicted optimal value	0.16^aB^	0.27^aA^
**Composite desirability**	**0.96**	**1**
Amylolytic activity (UI)	15.15 ± 0.76^a^	9.71 ± 0.40^b^
α-amylase activity (UI)	12.57 ± 0.28^a^	8.48 ± 0.20^b^
Malt loss	50.71 ± 0.00^a^	45.06 ± 0.00^b^
Malt yield	49.28 ± 0.00^b^	54.94 ± 0.00^a^
Kolbach Index (%)	0.14 ± 0.00^b^	0.21 ± 0.00^a^
Moisture content (%)	34.74 ± 0.13^a^	24.80 ± 0.00^b^
Water absorption capacity (%)	32.69 ± 0.69^a^	23.00 ± 0.20^b^
Water soaking pH before	9.60 ± 0.00^a^	9.59 ± 0.02^a^
Water soaking pH after	7.76 ± 0.06^b^	9.01 ± 0.01^a^
pH variation	1.84 ± 0.07^a^	0.58 ± 0.03^b^

The means (*n* = 3) ± standard deviations followed by the same letter in the same line indicate that the differences are not significant (*P* > 0.05).

Desirability is another parameter used to validate the mathematical model. It represents the percentage of reproducibility and robustness of a system. Using this parameter to validate a model requires a value > 0.5. This table shows that all the responses evaluated within each variety showed a good fit with the mathematical model. The composite desirability shows that the equations and optimal conditions are, respectively, 100 and 96% reproducible for the *Coca-sr* and *Atp-Y* varieties. These optimal conditions can be applied in agriculture to improve productivity and in malting. From the response optimization tests presented in Table 3, it appears that all experimental responses of both varieties did not differ significantly (*p* > 0.05) from those predicted by the system. The FAN content, proteolytic activity, and soluble protein content of the *Coca-sr* variety were significantly higher than those of the *Atp-Y* variety. The overexpression of the gene responsible for protease synthesis in the *Coca-sr* variety, the longer sprouting and ripening times, and the presence of proteases in more than one cell compartment could explain these differences. Moreover, the FAN content obtained with the *Coca-sr* variety is double the values obtained by Narziss and Back (66) in sprouted barley. The amylolytic activity also predicts the quality of the malt produced. Amylases are produced in the aleurone layer of cereals and break down starch into more digestible sugars. From the evaluation of amylolytic and α-amylolytic activites, it was found that the *Atp-Y* variety presented a value significantly (*p* < 0.05) higher than that of the *Coca-sr* variety. Tambo et al. (41) and Tsopbeng et al. (12) also reported a higher fluidizing capacity with the *Atp-Y* variety compared to the *Kassaï* and *Coca-sr* varieties. Milala and Addy (62) further reported that the enzymes produced during germination have an antagonistic effect on each other. Gujjaiah and Kumari (45) revealed inhibition of the alpha-amylolytic capacity of grains after 200 h of germination. These results suggest a combination of the two varieties in the formulation of cakes, energy-dense supplemental foods with acceptable viscosity. Similarly, a combination of these two varieties during malting would improve the quality of the malt and thus the beer. The evaluation of malt yield and malt loss of the sprouting maize grains revealed that mass loss is 50.71 and 45.06%, respectively, with an observed yield of 49.28 and 54.94% for *Atp-Y* and *Coca-sr*. This could be due to the long soaking time of the *Atp-Y* variety, which made the grain more brittle (67). In addition, the values of malt loss are higher than those reported by Embashu and Nantanga (7) which were between 11.00 and 30.40%. Another parameter that determines the malt quality is the Kolbach index. It is influenced by proteolytic activity and FAN content (68). This parameter varied from 0.21% for *Coca-sr* to 0.14% for *Atp-Y* variety. The difference observed could be explained by the losses of soluble proteins due to the long soaking time associated with high soaking temperature in the *Atp-Y* variety (69). Malt quality and malting process are also influenced by water holding capacity and moisture content of cereals grains. Table 3 shows that the moisture content and water holding capacity of the grains were 34.74 and 24.80% and 32.69 and 23.00% for the varieties *Atp-Y* and *Coca-sr*, respectively. This difference could be due to high soaking time and weak wall cells of the *Atp-Y* variety. Klang et al. (50, 51) also reported an increase in the moisture content of maize and rice grains with increasing soaking time. Many authors including Kolawole and Kolawole (70) have shown that the use of plant ash during soaking improves germination while reducing soaking time. Diffusion of these elements in the seed is influenced by the soaking time, the composition of the epidermis, and the soaking temperature. This will lead to a variation in pH as observed with both varieties (Table 3).

### 3.6. Physico-chemical and functional properties of *Atp-Y* and *Coca-sr* sprouted maize flours

#### 3.6.1. Chemical composition

The chemical value of the optimal flours was evaluated and recorded in Table 4. The water content influences the shelf life of the flour at room temperature and a content of <14% is favorable for long shelf life (71). The values obtained are 4.22 and 4.35% for *Atp-Y* and *Coca-sr*, respectively. They are lower than the values reported by Tambo et al. (8, 9), which ranged between 12 and 14% in maize meals (*Atp-Y* and *Coca-sr*). Indeed, authors have reported mobilization of the water contained in the seeds during the germination process (64). The drying time and temperature would also explain this difference. Protein content was not significantly (*p* < 0.05) affected by maize variety and optimal production conditions. The values obtained are higher than those of Tambo et al. (8). This could be explained by the destruction of insoluble complexes formed between tannins and proteins during germination (67). The values obtained are still below the recommended values (10–15%) for the formulation of supplementary feeds (72). The evaluation of ash and lipid content showed similar results in both varieties (1.00 and 6.18% for ash and lipid, respectively). The ash content is lower than those of Tambo et al. (8) and Kadher and Maheswari (73) which was 2.00 and 2.20% in Ragi and maize, respectively. This could be explained by a loss of soluble minerals during soaking due to the combination of temperature and soaking time. The lipid content is in the range of values recommended by Codex Stan (72). Tambo et al. (9) reported lipid contents of 7.25% in maize. Osman et al. (74) and Hussain and Burhanddin (75) explain this by the activity of lipolytic enzymes activated during germination and the use of lipids as an energy source. Fibers facilitate digestion and the fight against the occurrence of diseases related to metabolic disorders. They constitute the indigestible fraction of carbohydrates and are located in the wall. The *Atp-Y* variety presented the highest value (5.85%) for 1.02% for the *Coca-sr* variety. The destruction of the cell wall with the increase of germination and ripening time would explain this difference. Similarly, the activation of β-glucanase and β-amylase during germination would be at the origin of the destruction of soluble β-glucans and other insoluble carbohydrates of the wall leading to their loss (42, 47). The digestible carbohydrate content was also assessed. It is 75.29 and 79.51% for the *Atp-Y* and *Coca-sr* varieties, respectively. This difference can be explained by the high amylolytic activity observed in the *Atp-Y* variety (Table 3). The values obtained are in the range (70–80%) of the Codex Stan (72) recommendation for infant flours. The lowest starch and amylose contents were reported in the variety *Coca-sr*. The starch values are much lower than those reported by Tambo et al. (8, 9) which ranged from 46.02 to 66% in maize flour. Hussain and Burhanddin (75) reported that a decrease in starch content in sprouted cereals would be a consequence of hydrolysis to simple sugars and fermentable dextrins. The amylose/amylopectin ratio provides information on the rheological properties and functional applications of flour (2). A high ratio would predispose the flour to strong retrogradation. The difference observed in this study is related to variations in amylose content. These results indicate that the *Coca-sr* variety would be more applicable for bread making. The reduced sugar contents obtained in this study are 24 times higher than the values of Tambo et al. (8). This implies a possible application of these sprouted cereals in the sweetening of porridge and cakes. Furthermore, the energy density was evaluated and found to be 386.53 and 405.42 Kcal/100 g of *Atp-Y* and *Coca-sr* optimal sprouted flours, respectively. This difference is mainly related to their different chemical composition.

**Table 4:** Nutritional, mineral, and bioactive composition of *Atp-Y* and *Coca-sr* optimal germinated maize flours.

**Samples**	***Atp-Y***	***Coca-sr***
**Nutritional composition**		
Moisture (%)	4.22 ± 0.00^a^	4.35 ± 0.07^a^
Proteins (%)	7.95 ± 0.10^a^	7.46 ± 0.40^a^
Ash	1.00 ± 0.00^a^	1.00 ± 0.00^a^
Lipids (%)	6.17 ± 0.02^a^	6.18 ± 0.00^a^
Fibers (%)	5.85 ± 0.14^a^	1.02 ± 0.02^b^
Digestibles glucids (%)	75.29 ± 0.29^b^	79.51 ± 0.14^a^
Starch (%)	22.78 ± 0.99^a^	5.59 ± 1.06^b^
Amylose (% of starch)	15.57 ± 1.19^a^	1.64 ± 0.28^b^
Amylopectin (% of starch)	84.43 ± 1.19^b^	98.36 ± 0.28^a^
Amylose/amylopectin	0.18 ± 0.02^a^	0.02 ± 0.00^b^
Reducing sugar (% of glucids)	23.84 ± 1.04^a^	22.82 ± 0.11^a^
Energy content (Kcal)	386.53 ± 0.64^b^	405.42 ± 0.23^a^
**Mineral composition (mg/100 of DM)**		
Ca	778.50 ± 0.71^a^	779.00 ± 1.41^a^
Mg	71.80 ± 0.28^a^	70.90 ± 0.14^a^
Na	125.35 ± 1.22^a^	125.26 ± 1.08^a^
K	428.72 ± 2.10^a^	427.77 ± 0.76^b^
Cu	0.50 ± 0.02^a^	0.06 ± 0.00^b^
Ca/Mg	10.84 ± 0.03^a^	10.98 ± 0.00^a^
Na/K	0.29 ± 0.00^a^	0.29 ± 0.00^a^
Oxal/Ca	0.01 ± 0.00^a^	0.01 ± 0.00^a^
Oxal/Mg	0.12 ± 0.00^a^	0.13 ± 0.00^a^
Oxal/Cu	18.20 ± 0.78^b^	165.00 ± 21.23^a^
Phy/Ca	0.01 ± 0.00^a^	0.01 ± 0.00^a^
Phy/Mg	0.04 ± 0.00^a^	0.04 ± 0.00^a^
Phy/Cu	6.13 ± 1.48^b^	55.94 ± 7.19^a^
**Antinutritional and bioactive compounds**		
Phytates (mg/100g of DM)	3.05 ± 0.86^a^	3.05 ± 0.00^a^
Oxalates (mg/100 g of DM)	9.00 ± 0.00^a^	9.00 ± 0.00^a^
Trypsin inhibitors (mg/100 g of DM)	3.23 ± 0.12^a^	3.16 ± 0.16^a^
Cyanide (mg/100 g of DM)	1.48 ± 0.10^b^	2.19 ± 0.10^a^
Condensed tanins (mg CE/g of sample)	3.57 ± 0.11^a^	3.03 ± 0.25^a^
Hydrolysed tanins (mg CE/g of sample)	12.29 ± 0.51^b^	19.28 ± 1.83^a^
Phenols (mg GAE/g of sample)	49.33 ± 9.51^b^	71.18 ± 10.30^a^
Flavonoids (mg CE/g of sample)	27.19 ± 1.16^b^	91.18 ± 15.53^a^
Saponins (mg/100g of DM)	0.43 ± 0.03^a^	0.32 ± 0.06^b^

The means (*n* = 3) ± standard deviations followed by the same letter in the same line indicate that the differences are not significant (*P* > 0.05).

GAE, gallic acid equivalent; CE, catechin equivalent; DM, dry matter.

#### 3.6.2. Mineral composition and nutritional ratio

Minerals play a very important role in the body. They are involved in bone solidification, the transmission of nerve impulses, hemoglobin formation, respiratory phenomena, blood pressure regulation, and osmotics (76). They showed very high values of Ca (778.50 and 779.00 mg for *Atp-Y* and *Coca-sr*, respectively), Mg (71.80 and 70.90 mg for *Atp-Y* and *Coca-sr*, respectively), Na (125.35 and 125.26 mg for *Atp-Y* and *Coca-sr*, respectively), and K (428.72 and 427.77 mg for *Atp-Y* and *Coca-sr*, respectively). Cu is the least abundant mineral in these samples with a significantly high value (0.50 mg) in the *Atp-Y* variety compared to *Coca-sr* (0.06 mg). This would be related to the diffusion of this mineral from the plant salt to the seed during soaking. The results obtained with Ca, Mg, Na, and K are higher than those found by Dongmo et al. (2) in two maize varieties after soaking, roasting, and fermentation. The same is true for those obtained by Tambo et al. (8, 9). The activation of phytases and some digestive enzymes of negative polarity antinutrients such as oxalates during germination would be at the origin of the dissociation of the insoluble complexes formed between them and the ions, thus leading to their availability (77). Ndagire et al. (78) also reported an improvement in mineral content with increasing germination time. Nevertheless, the values obtained are all below the recommended Codex Alimentarus standard for K, Na, Ca, and Mg (72). The evaluation of mineral availability was also determined. The Ca/Mg and Na/K ratios show that there is no significant difference (*p* < 0.05) between the varieties. The Ca/Mg ratio higher than 1 shows a good availability of Ca and Mg in these two varieties. Similarly, the Na/K ratios of both varieties were found to be <1. This is related to their high K content and therefore makes them unsuitable for hypertensive patients (2). Phytates and oxalates are electronegative and can therefore chelate divalent cations present in foods thus reducing their availability (2). The molar ratios between these antinutrients and the different divalent cations evaluated were determined. The table shows that the phy/Ca, phy/Mg, oxal/Ca, and oxal/Mg ratios were not significantly (*p* < 0.05) influenced by the variety. The values obtained are lower than the norms (1 for phy/Mg and 0.17 for phy/Ca). These results suggest the availability of these ions and the contribution of the soaking-sprouting combination in improving the availability of ions and the destruction of antinutrients. The results obtained are also lower than those of Dongmo et al. (2) which ranged from 1.24 to 2.16 and 9.31 to 19.32 for phy/Ca and phy/Mg ratios, respectively. The phy/Cu and oxal/Cu ratios ranged from 6.15 to 55.94 and 18.20 to 165.00, respectively. The variety *Atp-Y* showed the lowest values. The high soaking time would be responsible for the loss of oxalates by solubilization.

#### 3.6.3. Anti-nutrients and bioactive compounds contents

The levels of phytates, cyanides, total phenols, and other anti-nutrient compounds were quantified. They have both negative and beneficial effects, notably by forming insoluble complexes with nutrients, thus reducing their digestibility and availability (77). Embashu and Nantanga (7) observed a positive correlation among phenols, tannins, and antioxidant properties. The oxalate and phytate contents are 9.00 and 3.05 mg/100 g, respectively, for the two varieties. The reported data are lower than those of Dongmo et al. (2) for phytates (39.50–76.28 mg/100 g). The activity of phytases produced during germination would explain this difference; as well as leaching out during soaking. These values are lower than the standard (250 mg). The trypsin inhibitor content was not influenced by the variety. The proportions obtained are in line with the results of Fotso et al. (63) who reported a 44% reduction of this antinutrient in soybeans after soaking with vegetable salt followed by cooking. Inhibition due to the pH of alkaline solutions, loss through leaching, and use for physiological needs of the plant during germination would explain these observations. Cyanogenic compounds are produced in cereals during germination. They have a protective effect on the growing plant. Tambo et al. (8, 9) reported a significant correlation (*r* = 0.965; *p* < 0.01) between enzyme activity and cyanide content.

The contents are 1.48 and 2.19 mg/100 g for *Atp-Y* and *Coca-sr*, respectively. The prolonged germination and maturation of *Coca-sr* would explain this difference. The values are still below the FAO/WHO standard of 10 mg HCN/100 g feed (79). Condensed tannins are mainly located in the plant wall in contrast to hydrolysable tannins which are located in the albumen of cereals. Condensed tannins were not affected by variety, unlike hydrolyzable tannins which were more pronounced (19.28 mg EC/g) in the *Coca-sr* variety. Varietal differences and leaching loss would explain this difference. Condensed tannin contents were lower than Embashu and Nantanga (7) which was 23.67 mg EC/g in red sorghum malt. Phenol, flavonoid, and saponin contents ranged from 41.33 to 71.18 mg GAE/g, 27.19 to 91.18 mg EC/g, and 00.32 to 0.43 mg/100 g, respectively. The variety *Atp-Y* had the lowest flavonoid and phenol content. Overexpression of polyphenol oxidases (PPO) during germination and leaching of these compounds are responsible for this variation (78). Embashu and Nantanga (7) obtained higher and lower levels of phenol (29.13 mg GAE/g) and flavonoids (6.21 mg EC/g) in red sorghum malt than ours, respectively. The optimal *Coca-sr* flour could therefore be recommended to patients suffering from metabolic disorders for its richness in bioactive compounds.

#### 3.6.4. Amino acid composition

Table 5 presents the amino acid composition of the optimal germinated maize flours. It can be seen from this table that four amino acids (Asn, Gln, Trp, and Cys) were not detected. This would be due to the methodology used, which led to their destruction. Indeed, acid hydrolysis leads to the destruction of Trp and Cys, and the transformation of Glutamine and Asparagine into their acid derivative. The *Coca-sr* variety showed the highest amino acid content. The high proteolytic activity, the destruction of insoluble complexes formed between antinutrients and proteins, thus improving their digestibility, and the long germination and maturation times are the causes. Essential amino acids were reported in higher quantities (3.60 mg/Kg) in the *Atp-Y* variety in contrast to the *Coca-sr* variety which presented a high content of non-essential amino acids (5.34 mg/Kg). Apolar essential amino acids like Leu (0.99 and 0.95 mg/Kg for *Atp-Y* and *Coca-sr*, respectively) were the most abundant in both varieties. This could be explained by the loss of polar soluble amino acids through leaching during soaking. In addition, hydrolysis of the structural proteins of the pectocellulosic wall consisting mainly of hydrophobic amino acids is also a consequence. Odukoya et al. (80) also reported a high content of Leucine in *S. aromaticum* spices. The proportions of aromatic amino acids obtained suggest a high chymotrypsin activity in the *Coca-sr* variety, whereas a high trypsin activity is reported in the *Atp-Y* variety. The high proportions of Glutamic and Aspartic acids in both varieties are explained by the transformation of Asparagine and Glutamine into these derivatives during acid hydrolysis. These amino acids are very important for the organism as they are involved in reactions catalyzed by transaminases (liver detoxification), neurotransmission, amylase, protease, and lipase activity (34). Poggiogalle et al. (81) reported high acidic amino acid content in the plants. Consumption of both varieties would provide very important Arginine for children (34). The low content of sulfur amino acids such as Methionine is explained by their use in physiological processes of the growing plant such as DNA synthesis or protein solidification (82). These observations suggest a combination with legume sources of sulfur amino acids when formulating supplemental feeds. The high contents of amino acids with antioxidant activity (Tyr, Ser, and Thr) have been reported in the *Coca-sr* variety. Protein quality depends on the content and proportion of different classes of amino acids (83, 84). The TEAAs/TNEAAs ratio of <1 in both varieties (0.67 and 0.69 for *Coca-sr* and *Atp-Y*) shows that these samples are not good sources of the so-called “Essential” amino acids. The high content of acidic amino acids is directly related to the acidic pH of the samples (80). The TEAAs/TAA ratio gives values higher than the 36% reported by Odukoya et al. (80) in *S. aromaticum* spices. The neutral amino acids with antioxidant activity (leucine, valine, and isoleucine) were the most dominant in these samples with a predominance in the *Coca-sr* variety. The Leu/Ile ratios are 2.75 and 2.88 for *Atp-Y* and *Coca-sr*, respectively. These values are lower than the 4.09 reported by Odukoya et al. (80). These results suggest that the consumption of these flours would pose less risk to the occurrence of Pellagra due to the negative interaction between the presence of leucine and tryptophan absorption.

**Table 5:** Amino acid compositions of *Atp-Y* and *Coca-sr* maize meals.

**Samples**	***Atp-Y***	***Coca-sr***
**Essential amino acids (EAAs) (mg/Kg)**		
His	0.21	0.32
Thr	0.41	0.48
Lys	0.4	0.26
Met	0.23	0.18
Val	0.46	0.46
Ile	0.36	0.33
Leu	0.99	0.95
Phe	0.54	0.59
**Non-essential amino acids (NEAAs)**		
Arg	0.42	0.41
Ser	0.46	0.46
Gly	0.39	0.38
Asp	0.67	0.72
Glu	1.55	1.67
Ala	0.67	0.56
Pro	0.6	0.66
Tyr	0.42	0.48
Total amino acids (TAA)	8.78	8.91
Total essential amino acids (TEAA)	3.6	3.57
Total non-essential amino acids (TNEAAs)	5.18	5.34
Total acidic amino acids (TAAAs)	2.22	2.39
Total basic amino acids (TBAAs)	1.03	0.99
Total neutral amino acids (TNAAs)	5.53	5.53
Total sulfur amino acids (TSAAs)	0.23	0.18
Total aromatic amino acids (TArAAs)	0.96	1.07
TEAAs/TNEAAs	0.69	0.67
Leu/Ile	2.75	2.88
% Cys in total sulfur amino acids	nd	nd

nd, not detected.

#### 3.6.5. Physical and functional properties of the optimal flours

Table 6 shows the physical and functional properties of the optimal flours. Properties such as pH, mass density, swelling rate, water retention capacity, and porosity are important in the choice of packaging and the choice of formulation to be used. The pH influences the feed intake, water holding capacity, and enzymatic activity of flours (2). It is influenced by the variety and the treatments applied. The values obtained were not significantly (*p* < 0.05) affected by variety and optimal production conditions. These values are higher than those of Tambo et al. (8, 9) which were between 6 and 6.50 in maize meal. This could be explained by the richness of these flours in acidic amino acids, and the release of organic acids during soaking and germination. The *Coca-sr* variety had the highest titratable acidity (3.25 meq NaOH/100 g).

**Table 6:** Physical and functional properties of the optimal flours.

**Samples**	***Atp-Y***	***Coca-sr***
**Physical properties**		
pH	5.56 ± 0.00^a^	5.57 ± 0.01^a^
Titrable acidity (meq of NaOH/100 g of DM)	1.50 ± 0.35^b^	3.25 ± 0.71^a^
Bulk density (g/ml)	0.47 ± 0.00^a^	0.42 ± 0.00^a^
Tapped bulk density (g/ml)	0.75 ± 0.01^a^	0.70 ± 0.00^a^
Porosity (%)	37.33 ± 0.24^a^	37.37 ± 0.18^a^
Hausner ratio	1.60 ± 0.00^a^	1.60 ± 0.00^a^
**Functional properties**		
Water retention capacity (%)	14.50 ± 2.12^a^	12.20 ± 0.28^b^
Swelling capacity (%)	57.19 ± 2.60^a^	58.16 ± 0.25^a^

The means (*n* = 3) ± standard deviations followed by the same letter in the same line indicate that the differences are not significant (*P* > 0.05).

The difference in basic and acidic amino acid composition explains this variation between the two varieties. Mass density is influenced by macronutrient content and particle size. Dongmo et al. (2) reported a reduction in mass density in maize meal with treatment. This is because hydrolysis of lipids, proteins, and carbohydrates leads to the formation of low molecular weight compounds and therefore less dense (85). Atlaw et al. (86) reported a reduction in particle size and therefore mass density of flours from Fenugrek after germination. These flours are suitable for the formulation of supplementary feeds and would also reduce packaging and transport costs (bulk density < 0.5). Porosity, Hausner ratio, and tapped density were not affected by the maize variety. Tapped density results are lower than those of Klang et al. (87, 88) obtained on maize and potato. Water holding capacity and swelling rate are influenced by starch content, protein content, and the nature of amino acids. They represent the hydration properties of flour. The swelling rate was not influenced by variety, while the water retention capacity was highest (14.50%) with the variety *Atp-Y*. This could be explained by the high starch content of this variety. Klang et al. (50, 51) showed a significant positive correlation between starch content and water-holding capacity. However, the results obtained are lower than those of Tambo et al. (8, 9), which ranged from 40 to 90% in maize flours. This could be explained by the hydrolysis of macronutrients that can give a high viscosity to flours during cooking by enzymes produced during germination (86, 87).

## 4. Conclusion

This study aimed to evaluate the influence of variety, soaking conditions (time, temperature, and ash concentration), germination, and maturation times on proteolytic activity, free amino nitrogen, soluble protein, and physico-chemical and functional properties of two maize varieties. It was found that proteolytic activity, soluble protein, and FAN contents were influenced by malting conditions and corn variety. The *Coca-sr* variety showed the highest FAN, soluble protein, and proteolytic activity. Under optimal conditions, both varieties required the same concentrations (1.678%) of vegetable salt. However, the amylolytic activity was more pronounced in the *Atp-Y* variety. The contents of protein, lipids, ash, water content, Ca, Mg, and Na were not influenced by the variety. Both varieties had relatively low levels of anti-nutritional compounds. The variety *Atp-Y* had the highest contents of essential amino acids. Glutamic and aspartic acids were the most abundant of the two varieties. With the exception of water retention capacity, all physical and functional properties showed similar values. This study shows a possible application of these malting conditions and maize varieties in the production of gluten-free cakes and beers, the improvement of digestibility of proteins contained in supplementary foods, as well as their sweetening and formulation of bakery products such as cakes with improved nutritional value.

## Data availability statement

The raw data supporting the conclusions of this article will be made available by the authors, without undue reservation.

## Author contributions

ST, JMK, OA, and HW: conceptualization, sample collecting, preparation, and designing of research work. ST, JMK, OA, DN, MK, AO, and JOK: execution of laboratory experiments and data collection. HW: supervision of the study. All authors: analysis of data and interpretation and preparation of the manuscript. All authors contributed to the article and approved the submitted version.
